# Harvest and decimation affect genetic drift and the effective population size in wild reindeer

**DOI:** 10.1111/eva.13684

**Published:** 2024-04-11

**Authors:** Thomas Kvalnes, Øystein Flagstad, Jørn Våge, Olav Strand, Hildegunn Viljugrein, Bernt‐Erik Sæther

**Affiliations:** ^1^ Norwegian Institute for Nature Research (NINA) Trondheim Norway; ^2^ Centre for Biodiversity Dynamics (CBD), Department of Biology Norwegian University of Science and Technology (NTNU) Trondheim Norway; ^3^ Norwegian Veterinary Institute Ås Norway; ^4^ Gjærevoll Center for Biodiversity Foresight Analyses Norwegian University of Science and Technology (NTNU) Trondheim Norway

**Keywords:** bottleneck, hunting, matrix population model, population dynamics, *Rangifer tarandus*, vital rates

## Abstract

Harvesting and culling are methods used to monitor and manage wildlife diseases. An important consequence of these practices is a change in the genetic dynamics of affected populations that may threaten their long‐term viability. The effective population size (*N*_e_) is a fundamental parameter for describing such changes as it determines the amount of genetic drift in a population. Here, we estimate *N*_e_ of a harvested wild reindeer population in Norway. Then we use simulations to investigate the genetic consequences of management efforts for handling a recent spread of chronic wasting disease, including increased adult male harvest and population decimation. The *N*_e_/*N* ratio in this population was found to be 0.124 at the end of the study period, compared to 0.239 in the preceding 14 years period. The difference was caused by increased harvest rates with a high proportion of adult males (older than 2.5 years) being shot (15.2% in 2005–2018 and 44.8% in 2021). Increased harvest rates decreased *N*_e_ in the simulations, but less sex biased harvest strategies had a lower negative impact. For harvest strategies that yield stable population dynamics, shifting the harvest from calves to adult males and females increased *N*_e_. Population decimation always resulted in decreased genetic variation in the population, with higher loss of heterozygosity and rare alleles with more severe decimation or longer periods of low population size. A very high proportion of males in the harvest had the most severe consequences for the loss of genetic variation. This study clearly shows how the effects of harvest strategies and changes in population size interact to determine the genetic drift of a managed population. The long‐term genetic viability of wildlife populations subject to a disease will also depend on population impacts of the disease and how these interact with management actions.

## 1 INTRODUCTION

An important consequence of harvesting populations is a change in their genetic dynamics (e.g. Allendorf et al., 2008; Kuparinen et al., 2016; Kuparinen & Merilä, 2007; Ryman et al., 1981; Waples, 2005). Harvest affects the genetic drift by changing demographic parameters, such as generation time, age structure, fecundity and survival, and the genetic variation in a population (Allendorf et al., 2008; Ryman et al., 1981; Sæther et al., 2009). High genetic drift over a long time can result in the loss of genetic variation, increase the probability of fixation of mildly deleterious mutations, and reduce the fitness of a population (Charlesworth, 2009; Kalinowski & Waples, 2002). The effective population size (*N*_e_) determines the extent of genetic drift in a population and therefore describes and predicts such changes (Kalinowski & Waples, 2002; Lande, 1985; Wright, 1931). As originally defined by Wright (1931), *N*_e_ is the size of a population which experience the same extent of genetic drift as an ideal population with constant size over time, random mating, non‐overlapping generations, and Poisson‐distributed family sizes. *N*_e_ is therefore a fundamental concept for assessment of long‐term genetic viability in conservation genetics (Franklin, 1980; Soule, 1980) and an important component of management strategies of threatened and vulnerable species (Frankham, 1995; Frankham et al., 2010; Kalinowski & Waples, 2002; Lande, 1995; Wang et al., 2016; Waples, 2002).

In wild populations, it is usually found that the effective population size *N*_e_ is less than the censused population size *N* (Frankham, 1995; Nunney, 1993; Nunney & Elam, 1994), with most estimates of *N*_e_/*N* in the range 0.1–0.5 (Frankham, 1995; Nunney, 1993, 1995; Nunney & Elam, 1994; Palstra & Fraser, 2012; Palstra & Ruzzante, 2008; Vucetich et al., 1997). *N*_e_ is determined by the demographic properties of a population, such as variation in reproduction and survival, generation time, and the sex ratio. Accordingly, a large degree of the variation in the *N*_e_/*N*‐ratio is due to differences in the life history of different taxa (Lee et al., 2011; Waples et al., 2013). For species with strong competition between males for access to mates, the ratio is expected to be at the lower end of the range (Lee et al., 2011; Myhre et al., 2017; Nunney, 1993). For instance, *N*_e_/*N* estimates from polygynous mammalian populations include 0.28 in moose *Alces alces* (Lee et al., 2019), 0.22 in northern elephant seals *Mirounga augustirostrus* (Nunney, 1993), 0.33 in bighorn sheep *Ovis canadiensis* (FitzSimmons et al., 1995), and 0.12 in red deer *Cervus elaphus* (Reed et al., 1986).

The minimum *N*_e_ for genetically viable natural populations, which prevents inbreeding depression and ensures lasting maintenance of genetic variation and the evolutionary potential, is much debated and proposals vary from 500 to 5000 (Allendorf & Ryman, 2002; Frankham et al., 2014; Franklin, 1980; Franklin & Frankham, 1998; Lande, 1995; Lynch & Lande, 1998). In such evaluations, the long‐term genetic viability is often assessed with a time frame of 40 generations, as scaling by generation time allows comparisons between taxa with different demography (Frankham et al., 2014). The criteria for minimum *N*_e_ are based on an assessment of the overall effect of genetic drift and the mutation rate (Franklin & Frankham, 1998; Wang et al., 2016), and should also include the effect of how quickly a population can adapt to changing environmental conditions (natural selection, Lynch & Lande, 1998). Franklin's (1980) well‐known proposal of *N*_e_ ≥ 500 has been criticized as being too low because it does not account for the influence of natural selection on the genetic variation (Lande, 1995; Lynch & Lande, 1998), it overestimates the proportion of new mutations that are neutral (Lande, 1995) and it ignores random effects from demographic, genetic, and environmental sources of stochasticity (Lynch & Lande, 1998). Recent assessments have found that a doubling of the minimum criterion to *N*_e_ ≥ 1000 is necessary to ensure long‐term genetic viability (Frankham et al., 2014). However, Lande (1995) assumes a lower rate of mutations that are close to neutral, an increased risk of fixation of mildly deleterious mutations, and also takes environmental and demographic stochasticity into account. Under these assumptions, he found that *N*_e_ ≥ 5000 is required to ensure long‐term genetic viability and evolutionary potential for adaptation to changed environmental conditions.

The literature also provides criteria for minimum *N*_e_ for short‐term genetic viability (defined as five generations). The most up‐to‐date estimate for such a criterion estimates that *N*_e_ must be greater than 100 (Frankham et al., 2014). The purpose of such a criterion is to ensure a low degree of inbreeding and the prevention of inbreeding depression over a short period. Over longer time periods, high genetic drift would be expected if the population size is this low, with negative consequences for the genetic variation and evolutionary potential. Hence, the most recent literature indicates that *N*_e_ should be 1000–5000 at minimum to ensure that natural populations have a high long‐term genetic viability (Allendorf & Ryman, 2002; Frankham et al., 2014; Lande, 1995; Lynch & Lande, 1998). The census population size must then often be greater than 2000–10,000, knowing that the ratio between *N*_e_ and *N* is commonly found to be in the range of 0.1–0.5 (Frankham, 1995; Nunney, 1993, 1995; Nunney & Elam, 1994; Palstra & Fraser, 2012; Palstra & Ruzzante, 2008; Vucetich et al., 1997).

Culling or intense harvesting is often used to monitor the occurrence and spread of wildlife diseases (Mysterud et al., 2020), and culling is also used to prevent the spread of diseases by removing or decimating populations or subpopulations to limit contact between animals (Uehlinger et al., 2016). Being invasive methods, they can have direct negative effects on the viability of monitored species. Thus, disease prevention or removal by culling or harvesting needs to be balanced against other management objectives and the long‐term population viability. Increased mortality or the decimation of a subpopulation will inevitably reduce the effective population size and result in increased genetic drift (Kuparinen et al., 2016; Ryman et al., 1981; Sæther et al., 2009). However, such effects could also result from diseases (Mysterud et al., 2023). Chronic wasting disease (CWD) is a fatal prion disease affecting cervids (Haley & Hoover, 2015). Infected individuals gradually accumulate an abnormal isoform of cellular prion proteins and die at advanced stages of the disease (Güere et al., 2020). Population level effects of the disease have been observed in white‐tailed deer (*Odocoileus virginianus*) and mule deer (*Odocoileus hemionus*) (DeVivo et al., 2017; Edmunds et al., 2016). Until recently, the disease was limited to North America and the Republic of Korea, but in 2016 it was discovered in Eurasian wild reindeer (*Rangifer tarandus*) and moose in Norway (Benestad et al., 2016). Management measures have been taken to prevent the spread of CWD in Europe and in 2017–2018 an entire subpopulation of more than 2000 wild reindeer in Norway was eradicated to prevent the spread into other subpopulations (Mysterud & Rolandsen, 2018). However, two more CWD cases have since been discovered in wild reindeer, one in 2020 and another in 2022, both in the Hardangervidda population in Norway (Tranulis et al., 2021). In the coming years, changes in the climate are expected to enhance the negative impacts of existing threats such as diseases and parasites to wild mammal populations (Jones et al., 2008; Mysterud et al., 2023). Thus, maintaining the genetic variation in wild populations is considered critical to ensure their long‐term persistence under present and future environmental conditions.

In this study, we demonstrate how to estimate *N*_e_ using demographic data from monitoring programmes and provide important knowledge on drivers of genetic drift that needs to be considered in nature management, for instance when handling outbreaks of diseases. Specifically, we estimate *N*_e_ of the wild reindeer population at Hardangervidda in Southern Norway and investigate the consequences of different harvest strategies and population decimation for changes in *N*_e_ and the genetic variation of the population (see Sæther et al., 2009 for a similar approach in moose). This population inhabits the largest continuous management area for wild reindeer in Norway, but is exposed to habitat fragmentation due to development of infrastructure and other human activities (Panzacchi et al., 2013, 2015). Its population size is regulated by harvesting, but the annual population growth rate also depends on weather conditions (Bargmann et al., 2019). Male biased harvest and reductions in population size have been suggested as management efforts to prevent the spread of CWD in this population (Mysterud et al., 2021). The analyses are performed in four steps. First, we develop and parameterize a matrix population model, using estimates of reproduction and survival for this population (Mysterud et al., 2020). This model is then used to estimate *N*_e_ of the population given the observed harvest rates for (1) the period 2005–2018 and (2) the year 2021. Second, we investigate the consequences of alternative harvest strategies for *N*_e_. The alternatives include variation in (1) the proportion of adult males (2.5 years or older) harvested, (2) the proportion of adult males and females harvested, (3) the proportion of calves harvested, and (4) the average proportional harvest rates in the period 2005–2018 (Lande et al., 1995). Third, we simulate changes in population size and allele frequency over a period of 100 years. We then investigate how the loss of genetic variation, estimated as heterozygosity, is affected by the severity of population decimations, harvest strategy, and the time a population is kept from growing. Finally, since the effect of population decimation will be much stronger on rare alleles than those that are common (Allendorf, 1986), we also perform a second set of simulations to investigate the loss of rare alleles following a bottleneck for different initial allele frequencies.

## 2 METHODS AND MATERIALS

### 2.1 Study system and data collection

The study was conducted using data from the wild reindeer population at Hardangervidda in Southern Norway (60°09′55″ N, 07°27′58″ E). Reindeer have a polygamous mating system, where there is strong competition between males for access to mates and reproduction is limited by the number of females available for mating. Females can become sexually mature in their second autumn (1.5 years old yearlings) and can give birth to one calf, in May (occasionally in early June) each year. The Hardangervidda population is subject to annual harvest prior to the rut in late summer or the beginning of autumn (August–September). Generally, hunters do not differentiate between female and male calves, and it is also difficult to determine the sex of yearlings (1.5 years old) during hunting. Thus, harvest quotas generally separate between calves (0.5 years old), females (2.5 years and older) or yearlings (females and males 1.5 years old), and free licenses (animal of any age and sex). The latter category is typically used to shoot adult males (2.5 years and older), as their size and status as trophy is considered attractive by hunters.

Four different annual surveys are performed throughout the year to monitor the population size and structure. First, a minimum estimate for the population size is made using flight transects during mid‐winter (January–March), where all observed groups of reindeer are photographed and counted. Second, the annual calf production is estimated using flight transects during summer (late June to mid‐July), where a subset of groups with females, calves, and yearling males are photographed and the ratio of calves to adult females and yearlings of both sexes is calculated. Adult males generally aggregate in separate groups in other areas at this time of the year. Third, data is recorded on the number of calves, yearlings, and adults of both sexes that are shot during the harvest (August–September). Finally, the population age and sex structure is estimated using ground surveys just after the harvest (September–October). At this time of the year the reindeer aggregate in groups with both sexes and can be classified into age and sex classes (calves, females, yearling males, and adult males).

### 2.2 Estimation of population sizes

The data from the four surveys enables us to estimate the annual population size (*N*), and age and sex structure just prior to the harvest (Kvalnes et al., 2024; see Supporting Information A, Figures A1–A3) using an integrated population model (Kéry & Schaub, 2012; Mysterud et al., 2020, 2023; Nilsen & Strand, 2018). This is a hierarchical change‐in‐ratio model with parameters estimated using Bayesian inference, and accounts for the available surveys having different coverages of the population from year to year. Population dynamics are modelled with a stage‐dependent matrix where females and males are separated into calves, yearlings, and adults. Annual (winter) survival is assumed common for all years and age categories, while fertility rates and summer survival rates of calves vary stochastically among years. We included survey data from 2005 to 2021. Priors were specified as uninformative, and the model was run with JAGS from R (Plummer, 2016) using the R‐package ‘jagsUI’ (Kellner, 2019). For more details, see Supporting Information A.

### 2.3 Population model

A two‐sex matrix population model is constructed to model the reindeer population. Females (*f*) and males (*m*) are classified as calves, yearlings, and adults, giving a population vector **N** = (*n*_*f*,0_, *n*_*f*,1_, *n*_*f*,2_, *n*_*m*,0_, *n*_*m*,1_, *n*_*m*,2_)^T^, where T indicates matrix transposition. The population is censused just prior to the harvest, giving a delayed post‐breeding census (Caswell, 2001). Annual harvest rates are given by the vector **h** = (*h*_*f*,0_, *h*_*f*,1_, *h*_*f*,2_, *h*_*m*,0_, *h*_*m*,1_, *h*_*m*,2_)^T^, where *h* is the proportion of a given age and sex class that is harvested. The population vector next year can then be estimated by **AN**, where the projection matrix **A** = (((**1** − **h**)**1** ^T^)^T^○A*). Here ○ indicates the Hadamard‐product, **1** is an all 1's column vector, and **A** = A* when all *h*'s are zero (no harvest). The expected projection matrix with no harvest (EA* = a*) can then be defined as

$$a^{*}=\left[\begin{array}{cccccc} 0 & \frac{1}{2}{\mathrm{qb}}_{f,1} & \frac{1}{2}{\mathrm{qb}}_{f,2} & 0 & \frac{1}{2}{\mathrm{qb}}_{m,1} & \frac{1}{2}{\mathrm{qb}}_{m,2}\\ s_{f,0} & 0 & 0 & 0 & 0 & 0\\ 0 & s_{f,1} & s_{f,2} & 0 & 0 & 0\\ 0 & \frac{1}{2}\left(1-q\right)b_{f,1} & \frac{1}{2}\left(1-q\right)b_{f,2} & 0 & \frac{1}{2}\left(1-q\right)b_{m,1} & \frac{1}{2}\left(1-q\right)b_{m,2}\\ 0 & 0 & 0 & s_{m,0} & 0 & 0\\ 0 & 0 & 0 & 0 & s_{m,1} & s_{m,2} \end{array}\right]$$

where *s*_*f*,*i*_ and *s*_*m*,*i*_ are the expected survival rates for age classes *i* = (0, 1, 2), *b*_*f*,*i*_ and *b*_*m*,*i*_ are the expected fecundities for age classes *i*, *q* is the primary sex ratio (the proportion of females at birth) and the fecundities are multiplied by ½ to account for sexual reproduction (Engen, Lande, & Sæther, 2005).

The expected female fecundity in each age class is given as the product of their expected survival and fertility, *b*_*f,i*_ = *s*_*f,i*_ *f*_*i*_, where the female fertility (*f*_*i*_) includes the summer survival for calves from birth to census just prior to the harvest. For males, the expected fecundity is given as $b_{m,i}=b_{m,i}^{*}c=s_{m,i}m_{i}c$, where *m*_*i*_ is the expected mating success for a male in age class *i* and *c* is a scaling constant. The male fecundity is limited by the availability of females for mating, but we assume that the proportion of males in the population does not limit female reproduction (see Supporting Information B). Thus, the male and female segments of the population have the same growth rate (*λ = λ*_*f*_ *= λ*_*m*_), which can be estimated by solving the classical Euler‐Lotka equation (Charlesworth, 1994) for the female population $q\sum_{i=0}^{\infty }l_{f,i}b_{f,i}\lambda_{f}^{-i-1}=1$, where $l_{f,i}=\prod_{j=0}^{i-1}s_{f,j}$ is the probability of survival for females from birth to age *i* and $l_{f,0}=1$. For a given mating success *m*_*i*_, the constant *c* in the equation for the male fecundity *b*_*m,i*_ can then be found by solving $c\left(1-q\right)\sum_{i=0}^{\infty }l_{m,i}b_{m,i}^{*}\lambda_{m}^{-i-1}=1$ when *λ*_*f*_ *= λ*_*m*_.

### 2.4 Effective population size

The effective population size per generation in age‐structured populations was defined by Engen, Lande, and Sæther (2005) as $N_{e}=\frac{N}{\sigma_{\mathrm{dg}}^{2}T}$, where *N* is the census population size, $\sigma_{\mathrm{dg}}^{2}$ is the demographic variance due to random variation between individuals in their ability to reproduce and survive (Lande et al., 2003), with subscript *g* to indicate that it includes a genetic component due to Mendelian inheritance in diploid species, and *T* is the average generation time. We use this result to estimate *N*_e_ for our reindeer population using demographic data.

The expected projection matrix E**A** = **a** has dominant eigenvalue *λ*, with right and left eigenvectors **u** and **v** defined by **au** = *λ* **u** and **v** ^T^ **a** = *λ* **v**. *λ* gives the multiplicative growth rate of the population in the average environment and if the eigenvectors are scaled such that $\sum u_{i}=1$ and **v** ^T^ **u** = 1, **u** is the stable age and sex distribution of the population and **v** gives the reproductive values for each age and sex class (Engen et al., 2009). The eigenvectors for the pure female or male projection matrix can be estimated similarly from the upper left or lower right submatrix, when the ½ factor on the fecundities is eliminated.

The average generation time of the population is estimated from the generation time for females and males, *T* = (*T*_*f*_ + *T*_*m*_)/2. Assuming that the expected sex ratio (*q*) is constant, $T_{f}=q\sum_{i=0}^{k}\left(i+1\right)l_{f,i}b_{f,i}\lambda^{-i-1}$ and $T_{m}=\left(1-q\right)\sum_{i=0}^{k}\left(i+1\right)l_{m,i}b_{m,i}\lambda^{-i-1}$, where *k* is the upper limit for the age. The demographic variance $\sigma_{\mathrm{dg}}^{2}$, with subscript g to indicate that it includes a genetic component due to random sampling of alleles in Mendelian segregation in diploid species, can be estimated from the averages and variances of the vital rates by (Engen, Lande, & Sæther, 2005)

$$\sigma_{\mathrm{dg}}^{2}\approx \sum_{l=0}^{5}\frac{\lambda^{-2}}{u_{l}}\sum_{\mathrm{ij}}\frac{\partial \lambda }{\partial a_{i,l}}\frac{\partial \lambda }{\partial a_{j,l}}\mathrm{Cov}\left(A_{i,l},A_{j,l}\right)Z_{l}.$$

Here *i* and *j* are defined from 0 to 5, *u*_*l*_ is element *l* in the stable age and sex structure (u) of the population, *Z*_*l*_ is set equal to the number of individuals *n*_*l*_ in age and sex class *l* = (0, 1, 2, 3, 4, 5) and $\partial \lambda /\partial a_{i,l}=v_{i}u_{l}$. Engen, Lande, and Sæther (2005) have shown that this approximation is accurate and provides detailed calculations for the (co)variances in the expression for $\sigma_{\mathrm{dg}}^{2}$ (see also Engen et al., 2010). We assume here that there are no covariances between survivals and fecundities in either sex.

### 2.5 Demographic and environmental variance

The annual total reproductive value for a population is given by *V*_*t*_ = **v** ^T^ **n**_**t**_ (Engen et al., 2009). At the stable age and sex structure **u**, *V*_*t*_ = *N*_*t*_, but fluctuations in age and sex structure between years cause temporary fluctuations in *N*_*t*_ around the deterministic growth. The total reproductive value does not display such fluctuations and grows according to the deterministic growth rate (Engen et al., 2009). The sequence of differences given by ln *V*_*t*+1_ − ln *V*_*t*_ has variance given by $\sigma_{e}^{2}+\sigma_{d}^{2}/N$, where $\sigma_{e}^{2}$ is the environmental variance and $\sigma_{d}^{2}$ is the demographic variance without the genetic component. Again, assuming that there is no covariance between survivals and fecundities, the demographic variance for the female subpopulation ($\sigma_{\mathrm{df}}^{2}$) can be estimated by $\sigma_{\mathrm{df}}^{2}=\lambda^{-2}\sum_{i=0}^{5}u_{i}\left(v_{0}^{2}\sigma_{\mathrm{bf},i}^{2}+v_{i+1}^{2}\sigma_{\mathrm{sf},i}^{2}\right)$ (Engen, Lande, Sæther, & Weimerskirch, 2005) and the total demographic variance $\sigma_{d}^{2}=\sigma_{\mathrm{df}}^{2}/q$ (Engen et al., 2007; Engen, Lande, & Sæther, 2005). Annual estimates of the environmental variance can then be estimated by subtracting the demographic variance in the variance of ln *V*_*t*+1_ − ln *V*_*t*_ and estimate $\sigma_{e}^{2}$ as the average of the annual estimates.

### 2.6 Parameter estimates

The population model was parameterized using estimates of average fertility for females (*η*), average summer survival for calves from birth to census (ϕ_1_) and survival for adults (ϕ_2_, excluding harvest mortality) from one census to the next (Table 1; Mysterud et al., 2020), remembering that census is set just prior to the harvest. These parameters were estimated for the Hardangervidda reindeer population by Mysterud et al. (2020) using the Bayesian hierarchical population model (see above) for the period 2008–2018. Their model did not differ between age or sex in the estimates for fertility and adult survival. Survival and fertility were assumed binomially distributed (Mysterud et al., 2020), where 1 is survival or the successful production of a calf. Thus, variances of the parameters can be estimated by $\sigma^{2}=\mu \left(1-\mu \right)$, where μ is the estimated average survival or fertility. To use these estimates for our population model we assume that the primary sex ratio (*q*) equals 0.5 (as shown by Loison & Strand, 2005 for the Hardangervidda population), that yearlings have a 10% reduction in fertility compared to adult females, and that annual survival (excl. harvest) is similar across age and sex classes. Generally, yearlings may have lower fertility rates relative to adult females in reindeer (Ropstad, 2000), but this varies between populations (Skogstad, 1985, 1989; Tyler, 1987). In the Hardangervidda population yearlings have been shown to have high fertility rates, at approximately 89% of adult fertility rates (Skogstad, 1985, 1989). The age‐specific fertilities (***η*** ^*^) were calculated taking the estimated fertility (*η*) to be the weighted mean with the mean number of yearlings and adults in the period 2005–2018 as weights. Older males which put a large effort into the rut may have a lower survival rate than younger males (Fancy et al., 1994; Mahoney & Virgl, 2003), but this effect is likely to be most evident in males that are 5–6 years or older (Reimers, 1983) and could not be included in our model. Still, in harvested populations, such as the Hardangervidda population, male biased harvest rates may often be a major component in the difference in annual survival between sexes (Fancy et al., 1994, Mahoney & Virgl, 2003).

**TABLE 1:** Estimates of the mean (*μ*), standard deviation (SD), and variance (*σ* ^2^) for female fertility (*η*), summer survival for calves (ϕ_1_), and survival from one census to the next (ϕ_2_, excluding harvest mortality) for the reindeer population at Hardangervidda.

Parameter	Estimate	
	*μ* (SD)	$\sigma^{2}$
Fertility for females (*η*)	0.649 (0.027)	0.228
Calf summer survival ($\phi_{1}$)	0.942 (0.032)	0.055
Adult survival ($\phi_{2}$)	0.934 (0.003)	0.062

*Note*: The variances were estimated by *μ*(1 − μ), while *μ* and SD are the mean and standard deviation of the posterior distributions (Table S2 in Mysterud et al., 2020). For fertility and calf summer survival the estimates are means of annual values for the parameters from the model.

The vital rates in the projection matrix (**a**) including harvest mortality were estimated by $b_{f,i}=\left(1-h_{f,i}\right)\phi_{2}\eta_{i}^{*}\phi_{1}$, $s_{f,i}=\left(1-h_{f,i}\right)\phi_{2}$ and $s_{m,i}=\left(1-h_{m,i}\right)\phi_{2}$. Thus, harvest reduces the estimated survivals and fecundities according to the proportion of the age and sex class harvested. The unscaled male fecundities $b_{m,i}^{*}$'s are estimated by $b_{m,i}^{*}=s_{m,i}m_{i}$, where *m*_*i*_ is the mating success for each age class. The strong competition between males may generally limit the access to females for young males and we have set the mating success for yearling males to half the mating success of adults such that **m** = (0, 0.5, 1). This degree of mating skew is within the range of what earlier has been recorded in reindeer (weighted mean ratio of yearlings to adults = 0.43, CI = [0.36, 0.90], Røed et al., 2005).

The variances in female and male survival are estimated as $\sigma_{\mathrm{sf},i}^{2}=s_{f,i}\left(1-s_{f,i}\right)$ and $\sigma_{\mathrm{sm},i}^{2}=s_{m,i}\left(1-s_{m,i}\right)$. Variances for female fecundities can be estimated by $\sigma_{\mathrm{bf},i}^{2}=\left(\sigma_{s_{f,i}}^{2}+s_{f,i}^{2}\right)\left(\sigma_{\eta *,i}^{2}+{\eta_{i}^{*}}^{2}\right)\left(\sigma_{\phi_{1}}^{2}+\phi_{1}^{2}\right)-s_{f,i}^{2}{\eta_{i}^{*}}^{2}\phi_{1}^{2}$, using standard formulas for the variance of the product of independent random variables. Assuming that the number of females which mate with males within a given age class is Poisson distributed, the variance in male fecundities can be estimated by $\sigma_{\mathrm{bm},i}^{2}=b_{m,i}/b_{f}\sum u_{f,i}\left(\sigma_{\mathrm{bf},i}^{2}+b_{f,i}^{2}\right)$ (Engen et al., 2010; Engen, Lande, & Sæther, 2005). Here $b_{f}=\sum u_{f,i}b_{f,i}$ and $u_{f,i}$ is the proportion of females in age class *i* at the stable age structure **u**_**f**_ for the pure female projection matrix. This estimate of the variance in male fecundity assumes that females choose to mate with males in proportion to the expected mating success for the age classes, without strong preferences for different males within the age classes. The relationship between the average and variance in male fecundity using this estimator is equal to $\sigma_{\mathrm{bm},i}^{2}=4b_{m,i}$, which is close to the relationship which earlier has been found in reindeer (e.g. Røed et al., 2005).

### 2.7 Estimated *N*_e_ with different harvest strategies

Two sets of analyses were performed to estimate the effects of different harvest strategies on the effective population size. First, the effective population size was estimated using the recorded average harvest rates in the population for the period 2005–2018 (excluding 2015, $h_{f,0}=0.105,h_{f,1}=0.078,h_{f,2}=0.129,h_{m,0}=0.136,h_{m,1}=0.181,h_{m,2}=0.156$, Supporting Information A, Table A1) and then using the harvest rates for the year 2021 (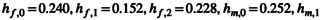 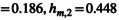, Supporting Information A, Table A1). Exclusion of the year 2015 was made because of uncertainties in the data regarding the population structure and the corresponding estimated harvest rates this year. However, mean harvest rates in the period did not change much by including 2015 and the results are not dependent on this exclusion. Second, the effect of different harvest strategies on *N*_e_ was analysed by choosing different harvest rates (*h*'s) for each age and sex class. Four types of harvest strategies were considered:

1. Increasing the proportion of adult males (2.5 years and older) harvested while keeping all other harvest rates at their 2005–2018 average.
2. Increasing the proportion of adult males and females harvested, while keeping all other harvest rates at their 2005–2018 average or scaling them to achieve a stable population (*λ* = 1).
3. Increasing the proportion of calves harvested, while keeping all other harvest rates at their 2005–2018 average or scaling them to achieve a stable population (*λ* = 1).
4. Increasing or reducing all the 2005–2018 average harvest rates while keeping the relative proportion of each age and sex class harvested constant.

These harvest strategies can be classified as constant or threshold harvest strategies (Lande et al., 2003). For a threshold harvest strategy, we set a limit *c* on the growth rate such that *λ* = *c* including harvest. The threshold harvest strategy thus means that we only harvest the population down to the threshold *c*, with no harvest when the population is below *c*. The constant harvest strategy on the other hand means that we set a harvest rate which is harvested irrespective of the effect on the population growth rate. We estimated *N*_e_ for both constant and threshold harvest, where *c* = 1 in the latter case for stable population dynamics. To achieve a given *λ* we perform a numerical approximation to estimate a scaling constant on the harvest rates based on *λ* in the female subpopulation (Supporting Information B). We also investigated effects on *N*_e_ if there was a changed degree of mating skew when the sex ratio was changed. This was only the case for harvest strategy 1, where we then estimated *N*_e_ using the original mating skew, **m** = (0, 0.5, 1), and a low degree of mating skew, **m** = (0, 0.9, 1). This is in accordance with studies showing that younger males may take a more active part in reproduction when the sex ratio becomes more skewed (e.g. Mysterud et al., 2003).

Statistical uncertainty in the estimates for generation time, demographic variance, and effective population size were quantified using parametric bootstrapping. We defined a beta distribution for each demographic rate with parameters $\alpha =\mu \left(\frac{\mu \left(1-\mu \right)}{{\mathrm{SE}}^{2}}-1\right)$ and $\beta =\alpha \frac{1-\mu }{\mu }$, where *μ* is the average and SE^2^ is the variance of the average (see Table 1). For population size we defined normal distributions with average *μ* and variance *σ* ^*2*^ estimated as the average and variance in the posterior distributions from the Bayesian hierarchical model (see above). For each parameter we made 1000 bootstrap replicates and estimated the 95% percentile confidence intervals (CI) from the bootstrap distributions.

### 2.8 Simulation of genetic drift

The effects of population decimation and different harvest strategies on the genetic variation, estimated as heterozygosity, were investigated by running simulations of a two‐dimensional diffusion approximation for logarithmic population size (ln *N*) and allele frequency (*p*) for a neutral locus (no selection). The initial allele frequency (*p*) was set to 0.5 and heterozygosity was calculated by *H* = 1 − (*p* ^2^ + (1 − *p*)^2^). The process for allele frequency (*p*) has infinitesimal mean of 0 and infinitesimal variance given by ${p\left(1-p\right)\sigma }_{\mathrm{dg}}^{2}/\left(2N\right)$, while the process for log population size (ln *N*) has infinitesimal mean given by ${r-\sigma }_{e}^{2}/2+\sigma_{d}^{2}/\left(2N\right)$ and infinitesimal variance given by $\sigma_{e}^{2}+\sigma_{d}^{2}/N$ (Engen, Lande, & Sæther, 2005), where *r* = ln *λ*. There is no infinitesimal covariance between *p* and ln *N*. Engen, Lande, and Sæther (2005) have shown that this diffusion approximation gives a precise and accurate description of the distribution of ln *N* and *p*.

The simulations took the census population size (*N* = 7720) and observed age and sex distribution (**U** = [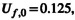 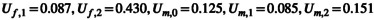]) at Hardangervidda in 2021 as their starting point and applied estimates of $\sigma_{e}^{2}$, $\sigma_{\mathrm{dg}}^{2}$, $\sigma_{d}^{2}$ and *λ* from the population model. Then they started with a population decimation to a census population size (*N*) of 200, 500, 1000, 2000, or 5000 animals after harvest, followed by a period of 0, 5, or 10 years where the populations were kept stable (λ = 1) using one out of four different harvest strategies (described below). Decimations were performed using the same harvest rate for all age and sex classes to avoid altering the age and sex distribution. The simulated populations were then allowed to grow without harvest until they reached the 2021 population size, where they were harvested using the average harvest rates from the period 2005–2018 scaled to yield stable dynamics (λ = 1). The four harvest strategies used in the simulations at reduced *N* were (1) a harvest rate of 90% of adult males (2.5 years and older), (2) harvest rates of 20% of adult males and females (2.5 years and older), (3) harvest rates of 50% for calves, and (4) average harvest rates in 2005–2018 for all age and sex classes. All other harvest rates in strategies 1–3 were set to the average harvest rates in 2005–2018, and calf, yearling, and adult female harvest rates were scaled to yield λ = 1. For harvest strategy 4, all harvest rates were scaled to yield λ = 1. The sizes of population decimations in the simulations were chosen to be relevant for nature management and have a good range of effects (from 35% to 97% of the population removed). Similarly, the periods with reduced population size were chosen to be relevant for disease management, where nature management might want to keep populations reduced to ensure sufficient time to remove a disease. The total number of scenarios was then 5 population decimations × 3 time periods × 4 harvest scenarios = 60 different scenarios. All simulations were run for 100 years forward in time with 1000 iterations, and the means and 50, 90, and 95% confidence intervals are calculated in the final time step. Temporal changes in population size and heterozygosity for the simulations of each scenario are shown in Supporting Information D.

The effects of population decimation on rare alleles were investigated using a second set of simulations. Then the loss of alleles following a bottleneck was simulated, using initial allele frequencies *p*_0_ = (0.01, 0.02, 0.05, 0.5). Through the bottleneck the census population size was reduced from *N* = 8000 to 200, 500, 1000, 2000, or 5000 individuals. All simulations were run for 1000 iterations and for each scenario the number of iterations which lose the focal allele is calculated.

All analyses were performed in the statistical software R version 4.3.3 (R Core Team, 2024).

## 3 RESULTS

The population of reindeer on Hardangervidda has a ratio *N*_e_/*N* = 0.239 (CI = 0.220–0.258) and an effective population size *N*_e_ = 1844 (CI = 1698–1999) given an estimated census population size *N* = 7725 (CI = 7594–7859) in 2021 and the average harvest rates in the period 2005–2018 (Table A1). However, the increased harvest rates in 2021, with an especially high harvest of adult males (44.8% of adult males harvested, Table A1), give *N*_e_/*N* = 0.124 (CI = 0.113–0.134) and *N*_e_ = 956 (CI = 877–1026). From Table 2 we can see that the decrease in *N*_e_ was caused by a doubling of the demographic variance ($\sigma_{\mathrm{dg}}^{2}$) while the generation time (*T*) only slightly decreased. The environmental variance ($\sigma_{e}^{2}$) was estimated to 0.007 and the demographic variance ($\sigma_{d}^{2}$, without the genetic component) was estimated to 0.575 in the period 2005–2018.

**TABLE 2:** Estimated mean and 95% confidence interval (CI) for reindeer on Hardangervidda in 2021 given that the population is harvested according to the average rates in the period 2005–2018 or according to the harvest rates in 2021.

Parameter	Harvest rate period			
	2005–2018		2021	
	Estimate	95% CI	Estimate	95% CI
Growth rate (*λ*)	1.028	(1.009, 1.047)	0.910	(0.893, 0.925)
Generation time (*T*)	6.42	(6.12, 6.77)	6.21	(5.98, 6.50)
Demographic variance ($\sigma_{\mathrm{dg}}^{2}$)	0.65	(0.63, 0.68)	1.30	(1.26, 1.36)

*Note*: Harvest rates in 2021 differed from the period 2005–2018 by an increased proportion of adult males being killed (see Table A1).

**FIGURE 1:**
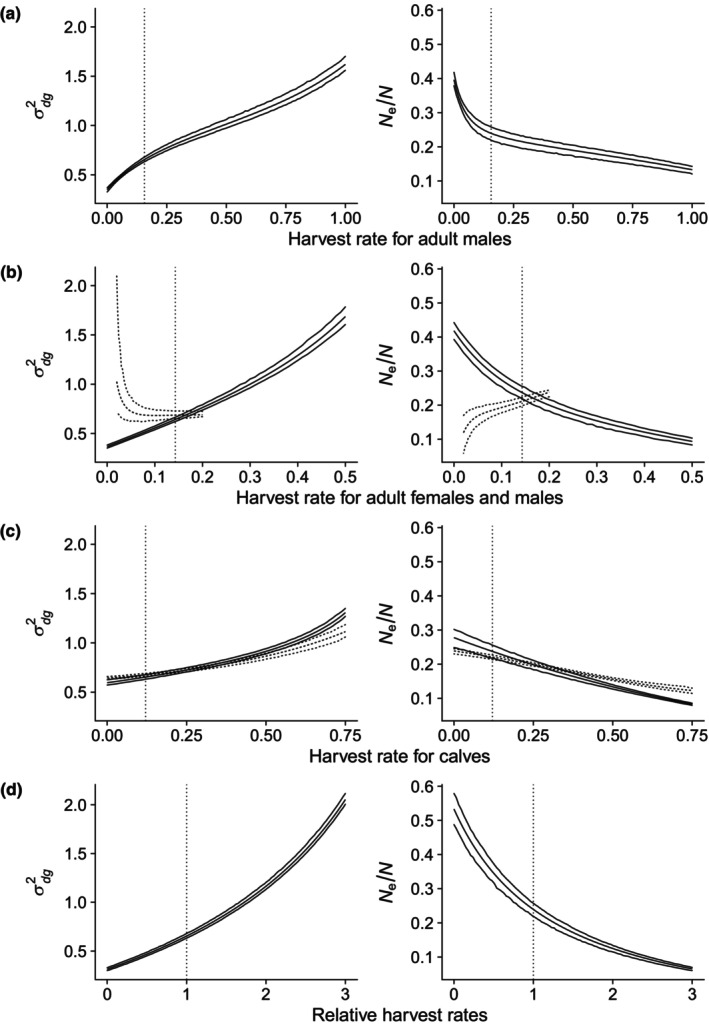
Demographic variance ($\sigma_{\mathrm{dg}}^{2}$) (left) and ratio of effective to census population size (*N*_e_/*N*) (right) for (a) different harvest rates of adult males (2.5 years and older), (b) different harvest rates for adult females and males, (c) different harvest rates for reindeer calves, and (d) different relative harvest rates for all age and sex classes. The solid lines show the results when the harvest rates of (a) calves, yearlings, and adult females, (b) calves and yearlings, or (c) yearlings and adults are kept constant at their averages ($h_{f,0}=0.105,h_{f,1}=0.078,h_{f,2}=0.129,h_{m,0}=0.136,h_{m,1}=0.181,h_{m,2}=0.156$). In (d) the harvest rates are given relative to the average rates, such that relative harvest rates of two equals a doubling of the average harvest rates. The dashed lines show the results when the average harvest rate of (b) calves and yearlings or (c) yearlings and adult females are scaled to λ = 1. The dotted vertical lines indicate the parameter values at the average harvest rate for (a) adult males, (b) adult males and females ($\left(h_{f,2}+h_{m,2}\right)/2=0.143$), (c) calves ($\left(h_{f,0}+h_{m,0}\right)/2=0.121$), and (d) all age and sex classes. The reindeer population at Hardangervidda in 2021 with a census size of *N* = 7725 and an observed age and sex distribution *U* = ($U_{f,0}=0.125,U_{f,1}=0.087,U_{f,2}=0.430,U_{m,0}=0.125,U_{m,1}=0.085,U_{m,2}=0.151$) is the basis for the calculations. The mating skew for male age classes was set to *m* = (0, 0.5, 1).

Increases in the harvest rates of all harvest strategies lead to a reduction of *N*_e_/*N* (Figure 1a–d, right panels), except when harvest rates for adults are offset by harvest rates for calves and yearlings to obtain stable population dynamics (Figure 1b). With this population model harvest rates above 21% for adults do not allow stable population dynamics to be achieved. We also find that the reduction in *N*_e_/*N* is lower when increased harvest rates for calves are offset by the harvest rates for yearlings and adult females to achieve stable population dynamics (Figure 1c). The differences in *N*_e_/*N* between harvest strategies are primarily caused by changes in genetic drift $\sigma_{\mathrm{dg}}^{2}$ (Figure 1a–d, left panels), with modification of these effects due to associated changes in generation time (*T*) (Supporting Information C, Figures C1–C4). The effect of a given harvest rate on the effective population size is also somewhat dependent on the census population size (Supporting Information C, Figures C5–C8). Male biased adult harvest rates (Figure 1a) will change the sex ratio of the population and may lower the mating skew. However, our results showed limited changes in *N*_e_/*N* when comparing the original (Figure 1a) to the low mating skew, with changes from 0.394 to 0.386 when *h*_*m*,2_ = 0, from 0.189 to 0.184 when *h*_*m*,2_ = 0.5 and no difference when *h*_*m*,2_ = 1. Hence, with diminishing numbers of older males, young males will have a larger proportion of the matings and mating skew has less effect on the estimate of *N*_e_.

**FIGURE 2:**
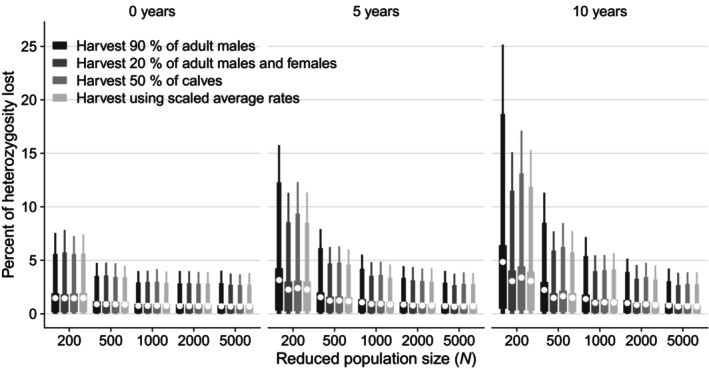
Loss of heterozygosity over 100 years after an initial population decimation where the population is kept stable at the reduced population size for 0, 5, or 10 years. Four different harvest strategies are applied in the period with reduced population size. (1) harvest 90% of adult males, (2) harvest 20% of adult males and females, (3) harvest 50% of calves, or (4) harvest using the estimated average rates. The population is kept stable in the period with reduced population size by scaling the average harvest rates of (1) calves, yearlings, and adult females, (2) calves and yearlings, (3) yearlings and adult females, and (4) all age and sex classes. *N*_0_ = 7725, *p*_0_ = 0.5, $\sigma_{d}^{2}$ = 0.581 and $\sigma_{e}^{2}$ = 0.007, and the population was allowed to grow without any harvest after the period with reduced population size until it reached *N*_0_, where it was kept stable by scaling all the average harvest rates. The average harvest rates are set to ($h_{f,0}=0.105,h_{f,1}=0.078,h_{f,2}=0.129,h_{m,0}=0.136,h_{m,1}=0.181,h_{m,2}=0.156$). The mating skew for male age classes was set to **m** = (0, 0.5, 1). The white point shows the average loss of heterozygosity, while 50%, 90%, and 95% confidence intervals are shown with error bars of different width.

**FIGURE 3:**
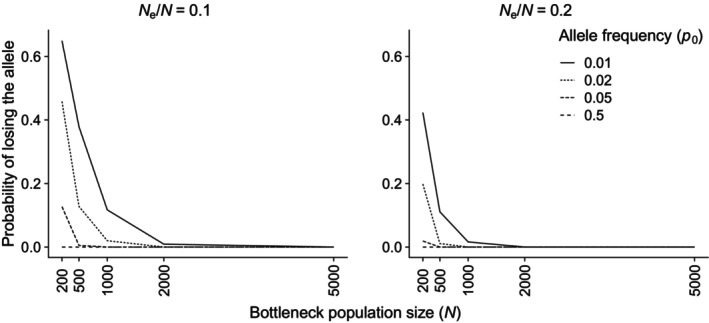
The probability of losing an allele following a population bottleneck caused by decimation to a census size of *N* = 200, 500, 1000, 2000, or 5000. The initial population size is set to *N*_0_ = 8000 individuals just prior to the bottleneck and initial allele frequencies *p*_*0*_ = 0.01, 0.02, 0.05, or 0.5. The ratio of effective to census population size (*N*_e_/*N*) is set to 0.1 (left panel) or 0.2 (right panel).

The simulations of genetic drift showed first that the loss of heterozygosity increases with the size of population decimations and the time which the population is kept at the reduced population size (Figure 2). Second, we see that there are differences between harvest strategies (Figure 2), with the most severe loss of heterozygosity found in the scenario with a large harvest rate for adult males. In Figure 2, we can see that a loss of more than 5% or 10% of heterozygosity is included in the 95% confidence intervals for many of the scenarios. Rare alleles are lost at a much higher rate for reduced population size less than *N* = 2000 (Figure 3). For *N*_e_/*N* = 0.1, around 40% of rare alleles (*p*_0_ = 0.01) are lost if the population is decimated to *N* = 500.

## 4 DISCUSSION

The effective population size (*N*_e_) of the reindeer population at Hardangervidda was closely linked to the choice of harvesting strategy (Figure 1). In 2019–2021, a large proportion of adult males were harvested as a preventive measure for reducing the potential transmission of CWD and for collecting samples to monitor CWD in the population (Table A1). Such high and skewed harvest rates resulted in *N*_e_/*N* = 0.124 in 2021 and an effective population size of 960 individuals. Thus, the population is just below the interval for recommended minimum *N*_e_ of 1000–5000 for long‐term genetic viability when harvesting adult males at this order of magnitude (Allendorf & Ryman, 2002; Frankham et al., 2014; Lande, 1995; Lynch & Lande, 1998). The average harvest rates in the period 2005–2018 (Table A1) are more representative of the harvest of this population in general and almost double the *N*_e_/*N* to 0.239. With these harvest rates *N*_e_ = 1849 for 2021 and the population is kept within the range of minimum for *N*_e_ in the literature which should ensure a high long‐term genetic viability. Thus, the biased harvest rates towards adult males in the period 2019–2021 had the consequence of increased genetic drift compared to the harvest rates normally seen for this population. Increased harvest rates generally also resulted in reduced *N*_e_ and higher genetic drift (Figure 1). This is a well‐known result of harvesting (Allendorf et al., 2008). Intuitively, this result can be understood as a consequence of fewer individuals contributing genetically to the next generation. Changing the mating skew when harvest is male biased only marginally reduced *N*_e_ beyond the large effect of increased harvest, which has also been found in moose (Sæther et al., 2009). However, at harvest rates that yield stable population dynamics, it turns out that you can reduce the decline in *N*_e_ by harvesting fewer adult females and young animals when you harvest calves (Figure 1c), or even increase *N*_e_ by harvesting less calves and yearlings when harvesting adult males and females (Figure 1b).

Estimation of *N*_e_ is possible using data on demographic factors and using genetic data on markers (Baalsrud et al., 2014; Nunney, 1995; Wang et al., 2016; Waples, 2023). Demographic data can provide estimates of current *N*_e_ in a population and enable detailed assessments of how various factors affect the genetic drift in the population (Engen, Lande, & Sæther, 2005; Waples, 2023). The challenge is that such estimators often require detailed knowledge of the population demography and dynamics, including fluctuations in population size, age structure, sex ratios, and vital rates. When using genetic markers, *N*_e_ can be estimated from heterozygosity excess or linkage disequilibrium from a single population sample or from temporal data on changes in allele frequency (Baalsrud et al., 2014; Nunney, 1995; Wang et al., 2016). Thus, the data for genetic methods are far less time‐consuming to collect than detailed demographic data where monitoring over several years is required to establish the age structure and vital rates. However, demographic methods are dynamic and therefore better suited for identifying the effect of various processes on *N*_e_. Comparisons of *N*_e_/*N* estimated using demographic and genetic methods show that results are often comparable (Baalsrud et al., 2014; Frankham, 1995; Nunney, 1995; Palstra & Fraser, 2012; Palstra & Ruzzante, 2008; Vucetich et al., 1997). In Canadian caribou or reindeer, Yannic et al. (2016) and Dedato et al. (2022) found genetic contemporary *N*_e_/*N*‐ratios to vary between 0.01 and 0.45 across different populations with different degrees of isolation and population size. We found ratios of similar magnitude using the definition of the effective population size per generation in an age‐structured population that was given by Engen, Lande, and Sæther (2005). This demographic method takes into account fluctuations in population size and fluctuations in the age and sex structure of the population (Engen, Lande, & Sæther, 2005) to enable detailed evaluations of different harvesting strategies by varying the different demographic rates in the population.

Several assumptions have been made to facilitate the estimation of *N*_e_. First, we assume that the demographic characteristics of the population do not depend on population size. Then there are no density‐dependent effects which would lead to decreased population growth with increasing population size. Myhre et al. (2016) have shown that such negative density‐dependent effects strongly influence the estimates of *N*_e_/*N*. However, with the population levels modelled here, there is little evidence that density dependence affects the population dynamics of wild reindeer on Hardangervidda (Bargmann et al., 2019). Allee effects are another effect of population size which could be important. These effects are seen as decreased population growth rate at low densities, for instance due to reduced ability of females to find a mate at low densities or through increased importance of stochastic effects (Lande, 1998). Such effects can occur as an unintended consequence of intensive harvesting (Myers et al., 1995) and can result in low population growth even in the absence of harvesting. In reindeer, rapid population growth has been found after introductions to new areas where populations have consisted of only a few individuals and also during re‐introductions of extinct populations (Aanes et al., 2000; Klein, 1968; Leader‐Williams et al., 1989; Thórisson, 1984). However, reduced growth rate at low population size has also been reported in reindeer (Wittmer et al., 2005). In addition, populations of reindeer have gone extinct after randomly skewed sex ratios and not being able to recover after harsh winters (three out of four introduced populations in Iceland, Thórisson, 1984). Secondly, we assume that this population is closed and has a continuous geographical distribution. This means that we do not consider immigration into the population or account for spatial structure in the distribution of animals on the Hardangervidda. Some limited number of immigrants to the population is likely from neighbouring populations (Røed et al., 2014). However, there has been found significant differentiation between these populations (Røed et al., 2014), and results from animals monitored with satellite transmitters suggest that there is a rather limited degree of migration between the reindeer populations around Hardangervidda (Beyer et al., 2016; Panzacchi et al., 2013). The development of infrastructure and other human activities are important factors which cause habitat fragmentation and limit animal movements (Panzacchi et al., 2013, 2015). Third, we assume that all bucks in an age class have an equal probability of achieving a mating. Fourth, we assume that the environment does not change over time, but rather fluctuates around the same level with no temporal change in the magnitude of fluctuations.

The population size of wild reindeer on Hardangervidda is estimated based on the four surveys in the annual monitoring of the population and is associated with some uncertainty (Mysterud et al., 2020, 2023; Nilsen & Strand, 2018). A harvesting strategy based on overestimation of the actual population size will result in an unintentionally increased risk of loss of genetic variation and population decline (Lee & Sæther, 2022; Wiedenmann & Jensen, 2018). Thus, the uncertainty in the annual population estimates should be considered when choosing a harvesting strategy for wild reindeer (Engen et al., 1997; Sæther et al., 2010; Tufto et al., 1999) and interpreting its effect on the effective population size for long‐term genetic viability. The monitoring does not allow the full age structure of the population to be estimated, as it is not possible to distinguish between age and sex classes in the field beyond what is already recorded. This is a common issue, not the least in nature management applications, which sets the limits for the complexity of the population model which could be used in this study (see Section 2). However, as also harvesting quotas are set with the same sex and age classes used in the population model, the simplified age structure will yield reasonable estimates for the processes included in the study, which also are relevant for nature management decisions. In a comparison of complex and simplified age structure in population models for moose, Lee et al. (2019) found that *N*_e_/*N* was essentially unchanged by increasing the number of age classes. However, as less variation in vital rates was explained by the simplified age structure, the estimate of demographic variance was slightly inflated while the estimated generation time was deflated (Lee et al., 2019). The magnitude of such effects is difficult to predict but will depend on the degree to which the simplified age structure captures the complexity of the vital rates.

In applied conservation genetics, a goal of preserving at least 90% (Frankham et al., 2010) or 95% (Allendorf & Ryman, 2002) of the genetic variation (often calculated as heterozygosity) in a population over 100 years is often used. The time interval of 100 years is generally in between the short‐term (5 generations) and long‐term (40 generations) time periods that are regularly used when assessing genetic viability using effective population size (Frankham et al., 2014). It has been chosen based on an expectation that the environment will not change significantly during a period of this length, such that an assumption of a constant fluctuating environment can be used. Our simulations of the loss of heterozygosity over 100 years at various reductions in population size show that we can risk higher losses than both these criteria in an isolated population under several scenarios (Figure 2). In particular, a high harvest rate of adult males (90%) results in a risk of losing more than 5% of the heterozygosity even at *N* = 2000 if the population is kept low for 10 years (Figure 2). When assessing the expected loss of genetic variation after a population reduction, both the harvesting strategy and the expected time period with a low population size must be accounted for. In our simulations, less than 5% loss of genetic variation (measured as loss of heterozygosity) is possible if the time the population is kept low is short and a harvest strategy that gives a high *N*_e_/*N* is applied. The results imply a period without harvesting to allow the population to grow, which will be long in case of a large population decimation. Harvest during periods of high population growth will increase the genetic drift and the loss of genetic variation above our estimates. The simulated scenarios are considered relevant examples, but no attempt has been made to address which harvest strategies that will optimize the population's genetic viability.

For a period of 0–5 years with a reduced population size where up to 10% loss of heterozygosity is accepted, our results indicate that there is a 95% certainty of achieving this goal with a reduced population size of *N* = 500 animals after harvest (Figure 2). Even with *N* = 200 after harvest, there will be 95% certainty of losing less than 10% loss of heterozygosity when the population immediately grows in the years after the population decimation. However, these results strongly depend on the assumption that there are no Allee effects, which is likely to be questionable for decimations of this extent. In addition, bottlenecks such as this will have a larger effect on the diversity of alleles than on heterozygosity because of an increased loss of rare alleles (Figure 3; Allendorf, 1986). Rare alleles contribute relatively little to heterozygosity and the loss of rare alleles is therefore not well captured by this metric. Still, such losses can have a large effect on the genetic viability of a population and its long‐term evolutionary potential (Allendorf et al., 2008). Heterozygosity is a good measure of a population's ability to evolve immediately after a bottleneck, but over longer periods of time, a population's ability of adaptive evolution will also depend on the diversity of alleles in the population (James, 1971; Robertson, 1960). Estimates for the loss of genetic variation measured as heterozygosity must therefore be seen as a conservative estimate that to a small extent includes the loss of rare alleles, especially in the event of a substantial population reduction (*N* < 1000; Allendorf & Ryman, 2002). The reduction in the evolutionary potential over a longer period of time could thus be greater than what is shown by the loss of heterozygosity in the various scenarios that have been modelled.

The management of wild reindeer in Norway is now faced with a situation where long‐term genetic viability is threatened by the outbreak of CWD (Benestad et al., 2016). CWD is fatal to individuals who become infected, and an extensive outbreak can have a negative impact on the future growth rate of the population and possibly cause a decrease in population size (DeVivo et al., 2017; Edmunds et al., 2016; Haley & Hoover, 2015). The risk of transmission of CWD to other species of cervids or domestic animals may also be present (Moazami‐Goudarzi et al., 2021). Accordingly, the consequence of CWD infection in the population on Hardangervidda and the impact of suggested management strategies must be based on updated knowledge and carefully weighed against their likely impact on the population's genetic viability. Severe population reductions or the removal of populations can be the last resort to stop an outbreak (Mysterud et al., 2020, 2021; Mysterud & Rolandsen, 2018; Uehlinger et al., 2016). However, bottlenecks and reduced population sizes kept over longer time periods could have very negative consequences for the long‐term genetic viability of wild populations due to genetic drift and loss of rare alleles. Results in a recently conducted study of the Nordfjella wild reindeer population in Norway found that there were polymorphisms in the gene (PRNP) that codes for the prion protein, some of which differed in their risk for testing positive for CWD (Güere et al., 2020). Similar results have also been found in other species of cervid and domestic animals (reviewed in Moazami‐Goudarzi et al., 2021). This emphasizes the importance of maintaining genetic variation in wild populations to ensure that their evolvability remains high. Thus, for disease management there is a need to develop detailed models where the effect on *N*_e_ of different harvest strategies can be assessed against the risk of further spread of an infection. In this way, better harvest models for disease surveillance and control can be developed, which maximize the effective population size, while at the same time meeting the management's need for infection tracking and minimizing the negative consequences of further outbreaks.

## CONFLICT OF INTEREST STATEMENT

The authors have no conflict of interest to declare.

## Supporting information


Data S1

## Data Availability

The data on population sizes and harvest rates, as well as R codes for analyses are available at the Dryad Digital Repository (https://doi.org/10.5061/dryad.brv15dvh6).
