# Supplementary material for: Harvest and decimation affect genetic drift and the effective population size in wild reindeer
Source: Evol Appl. 2024 Apr 11;17(4):e13684. doi: 10.1111/eva.13684 (PMC11009432; doi:10.1111/eva.13684)
Supplement: Supplementary file 1 — Data S1 [file EVA-17-e13684-s001.docx]

# Supplementary material for “Harvest and decimation affect genetic drift and the effective population size in wild reindeer”

Kvalnes, T., Flagstad, Ø., Våge, J., Strand, O., Viljugrein, H., Sæther, B.-E. 2024. Harvest and decimation affect genetic drift and the effective population size in wild reindeer. *Evolutionary Applications.*

## A. Harvest rates and population sizes

### Table and figures

Table A1: Mean +/- standard error (SE) harvest rates in the periods 2005-2018 (excl. 2015) and 2021 for the reindeer population at Hardangervidda in Norway. Exclusion of the year 2015 was made because of uncertainties in the data regarding the population structure and the corresponding estimated harvest rates this year. The harvest rate is given as the proportion of each age and sex class which is harvested annualy. Adults are all animals of age 2.5 years or older. The harvest is conducted in the autumn just prior to the rut, when calves are 3-4 months old.

|  | Harvest rates | | |
| --- | --- | --- | --- |
|  | Years 2005-2018 | | Year 2021 |
|  | Mean ± SE | Range | Estimate |
| *Females* |  |  |  |
| Calves | 0.105±0.018 | 0.037-0.264 | 0.240 |
| Yearlings | 0.078±0.013 | 0.032-0.196 | 0.152 |
| Adults | 0.129±0.017 | 0.042-0.249 | 0.228 |
| *Males* |  |  |  |
| Calves | 0.136±0.018 | 0.063-0.283 | 0.252 |
| Yearlings | 0.181±0.027 | 0.048-0.352 | 0.186 |
| Adults | 0.156±0.018 | 0.079-0.302 | 0.448 |


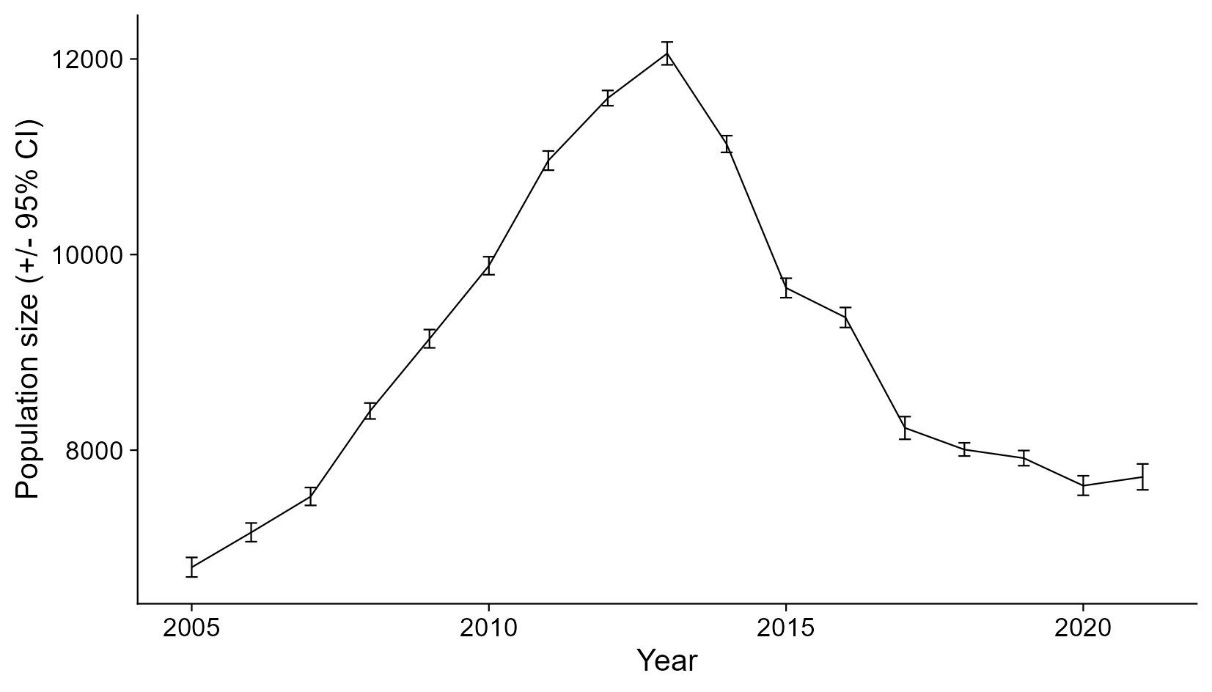


Figure A1: Estimated total post-breeding population size (average ± 95 % credible interval) for each year in the population of reindeer at Hardangervidda. Population sizes were estimated from population surveys using a Bayesian integrated population model. The census for the population is set in the autumn just prior to the annual harvest, when calves are 3-4 months old.


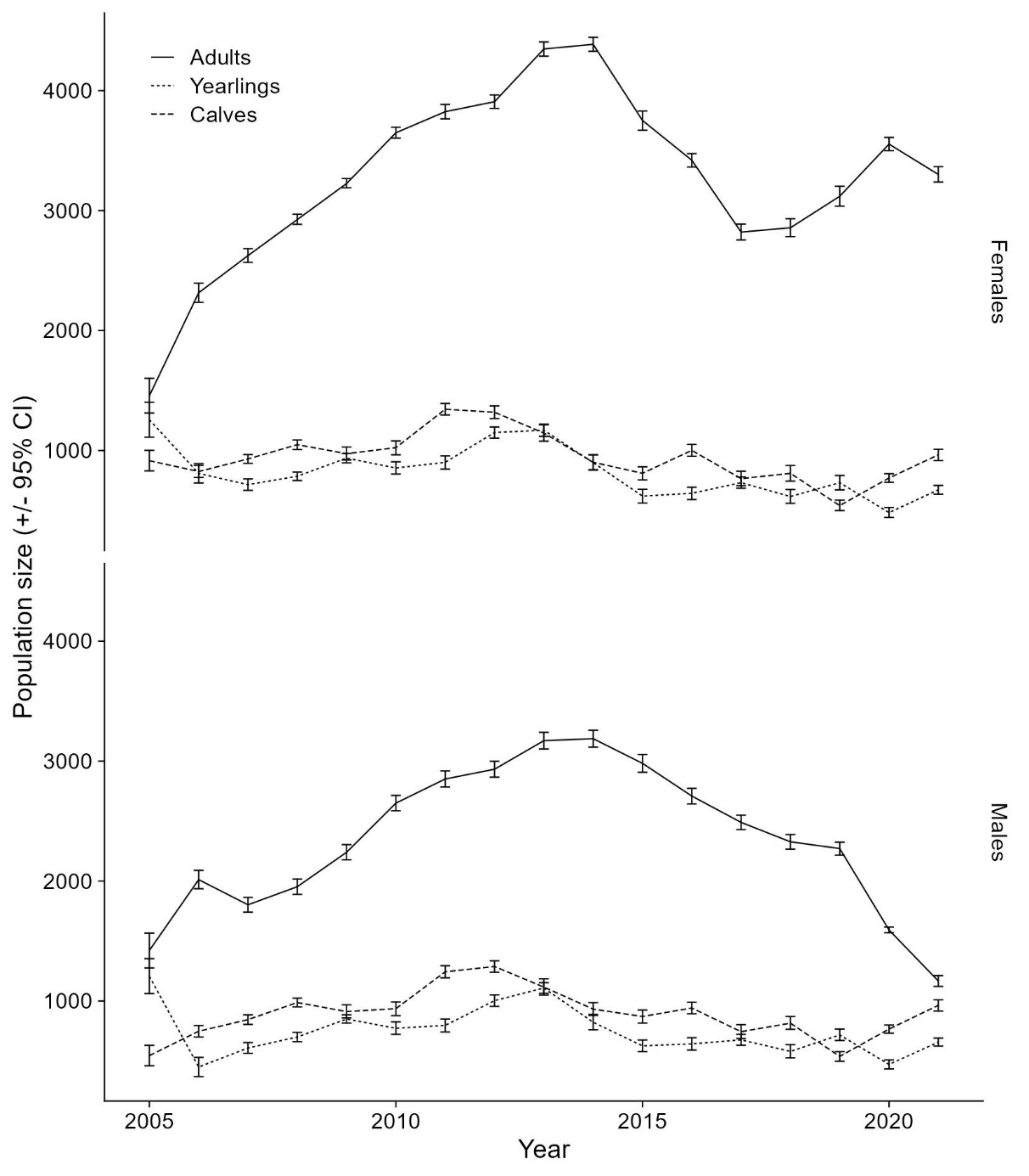


Figure A2: Estimated post-breeding population size (mean ± 95 % credible interval) for each age and sex class in the population of reindeer at Hardangervidda. Population sizes were estimated from population surveys using a Bayesian integrated population model. The census for the population is set in the autumn just prior to the annual harvest, when calves are 3-4 months old.


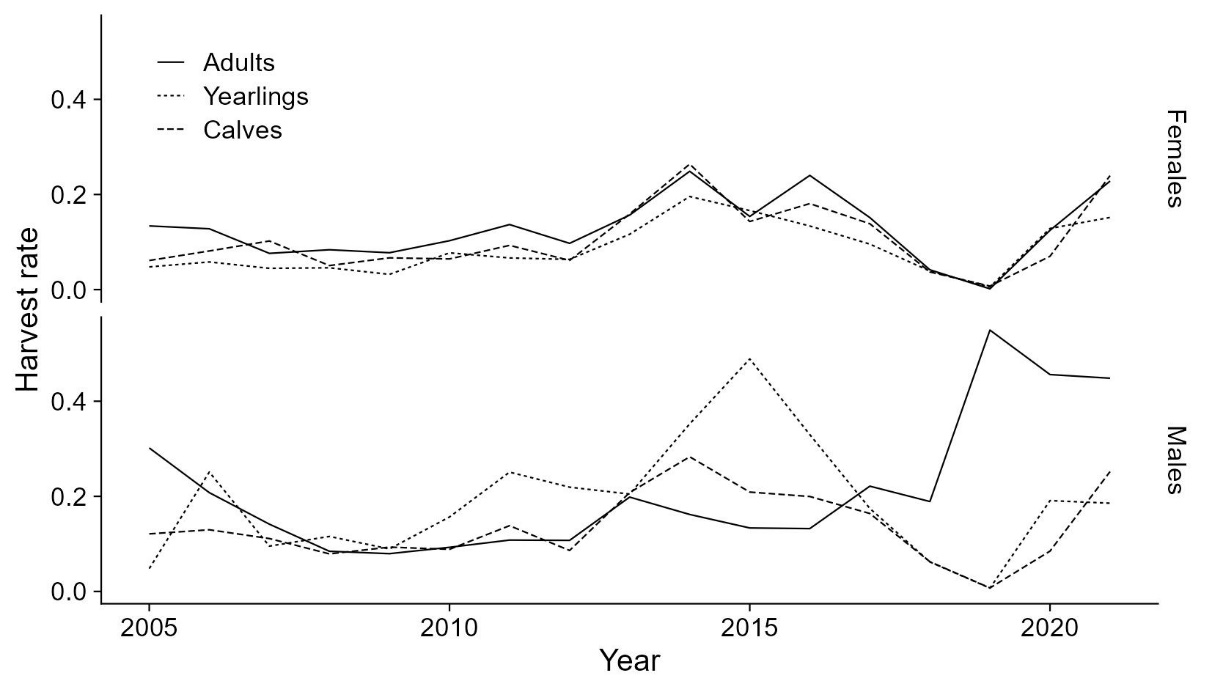


Figure A3: Estimated annual harvest rates for each age and sex class in the population of reindeer at Hardangervidda. The estimates are based on the reported number of animals harvested and the estimated post-breeding population size of each age and sex class.

### Estimation of population sizes

The Bayesian integrated population model assumes autumn counts reflect the demographic composition in the total population. The original model (Nilsen and Strand 2018) was slightly modified to incorporate demographic composition counts in 2020, when all yearlings, both males and females, were summed together with adult females (Mysterud et al. 2023). After 2018, there were no winter counts for Hardangervidda. For 2019 and 2021, total summer counts were included as a minimum count of the pre-harvest population, due to a large proportion of the adult males being covered by the summer counts. Priors were specified as uninformative, using uniform distributions (0–1) for probabilities and wide normal distributions for the initial population sizes. After a burn-in period of 50 000 and thinning by three, 750 000 samples from three MCMC chains were used for the posterior distributions. Convergence was assessed by visual inspection of MCMC chains and the Gelman-Rubin statistics. For data and R-script, see Viljugrein (2023).

### References

Mysterud, A., Viljugrein, H., Andersen, R., Rauset, G.R., Reiten, M.R., Rolandsen, C.M, Strand, O. 2023. An infectious disease outbreak and increased mortality in wild alpine reindeer. Ecosphere, 14: e4470.

Nilsen, E.B., Strand, O. 2018. Integrating data from multiple sources for insights into demographic processes: Simulation studies and proof of concept for hierarchical change-in-ratio models. PLoS ONE, 13: e0194566.

Viljugrein, H. 2023. Data and Figure-Scripts for the Paper ‘An Infectious Disease Outbreak and Increased Mortality in Wild Alpine Reindeer’. Zenodo. doi: 10.5281/zenodo.7624490

## B. Projection matrix – female limited growth rate

If we assume that the population is only limited by the number of females available for mating and that there are always sufficient males to mate with all females. Then the expected projection matrix without harvest ($E\mathbf{A}_{\mathbf{f}}^{\mathbf{*}}$) can be given as

$E\mathbf{A}_{\mathbf{f}}^{\mathbf{*}}=\left[ \begin{matrix} E\mathbf{A}_{\mathbf{f,ff}}^{\mathbf{*}} & E\mathbf{A}_{\mathbf{f,mf}}^{\mathbf{*}} \\ E\mathbf{A}_{\mathbf{f, fm}}^{\mathbf{*}} & E\mathbf{A}_{\mathbf{f, mm}}^{\mathbf{*}} \end{matrix} \right]= \left[ \left. \begin{matrix} 0 & {qb}_{f,1} & {qb}_{f,2} & 0 & 0 & 0 \\ s_{f,0} & 0 & 0 & 0 & 0 & 0 \\ 0 & s_{f,1} & s_{f,2} & 0 & 0 & 0 \\ 0 & {(1-q)b}_{f,1} & {(1-q)b}_{f,2} & 0 & 0 & 0 \\ 0 & 0 & 0 & s_{m,0} & 0 & 0 \\ 0 & 0 & 0 & 0 & s_{m,1} & s_{m,2} \end{matrix} \right] \right.$ ,

where the non-null matrix elements are the expected annual fecundities for females ($b_{f,i}$) and the annual survivals for both sexes ($s_{f,i}$ og $s_{m,i}$). For this model the number of males in the population will not affect the population growth rate. This is a purely demographic model which do not consider genetic contributions of males to the next generation.

## C. Effects of harvest on the effective population size


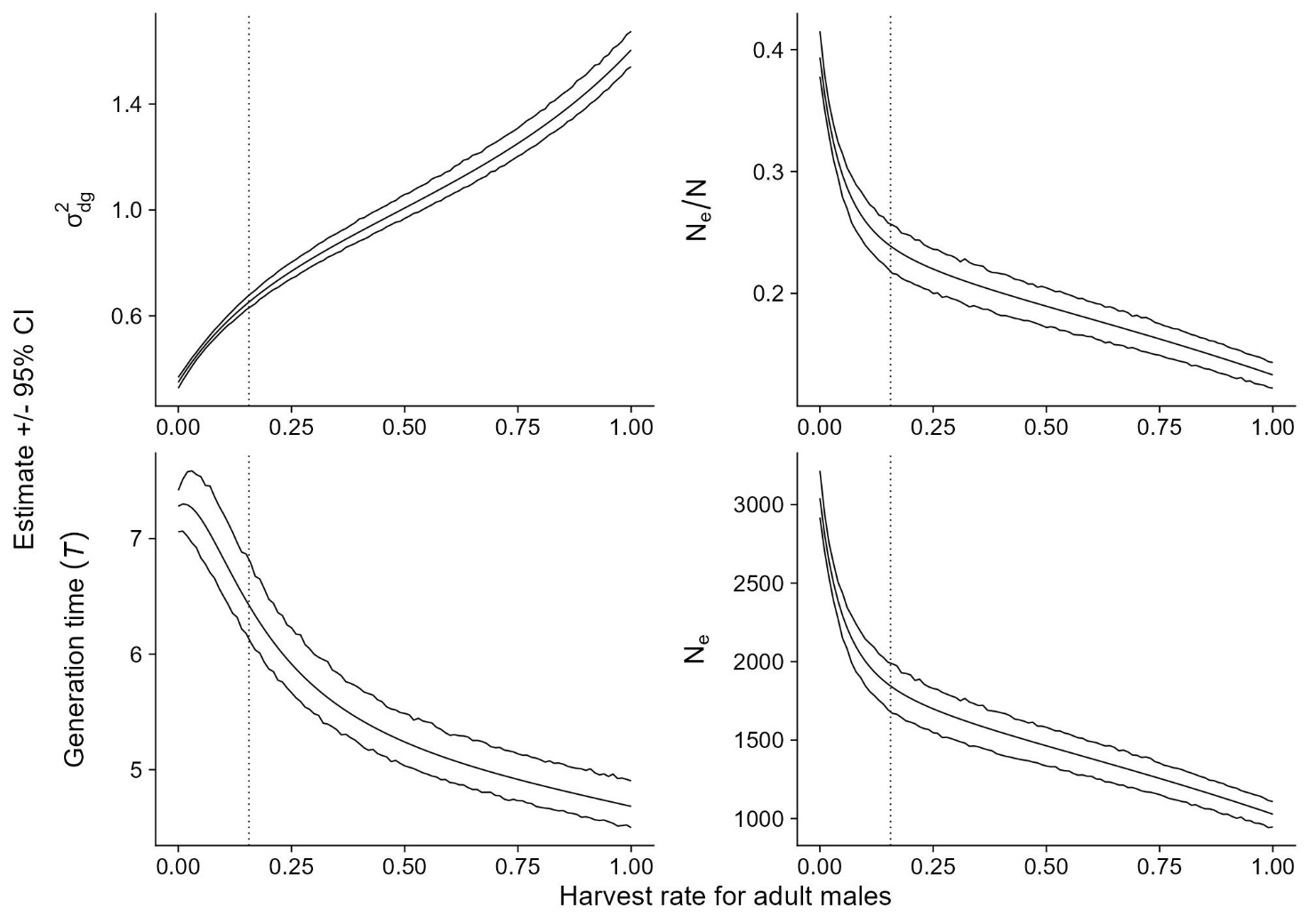


Figure C1: Demographic variance ($\sigma_{dg}^{2}$), generation time (T), ratio of effective to census population size (N_e_/N) and N_e_ (given N = 7725 in 2021) for different harvest rates of adult males (2.5 years and older). The harvest rates for all other age and sex classes are held constant at their average ($h_{f,0}=0.105, h_{f,1}=0.078, h_{f,2}=0.129, h_{m,0}=0.136, h_{m,1}=0.181$), which give a growth rate λ = 1.028. The dotted vertical line indicates the parameter values at the average harvest rate for adult males ($h_{m,2}=0.156$).


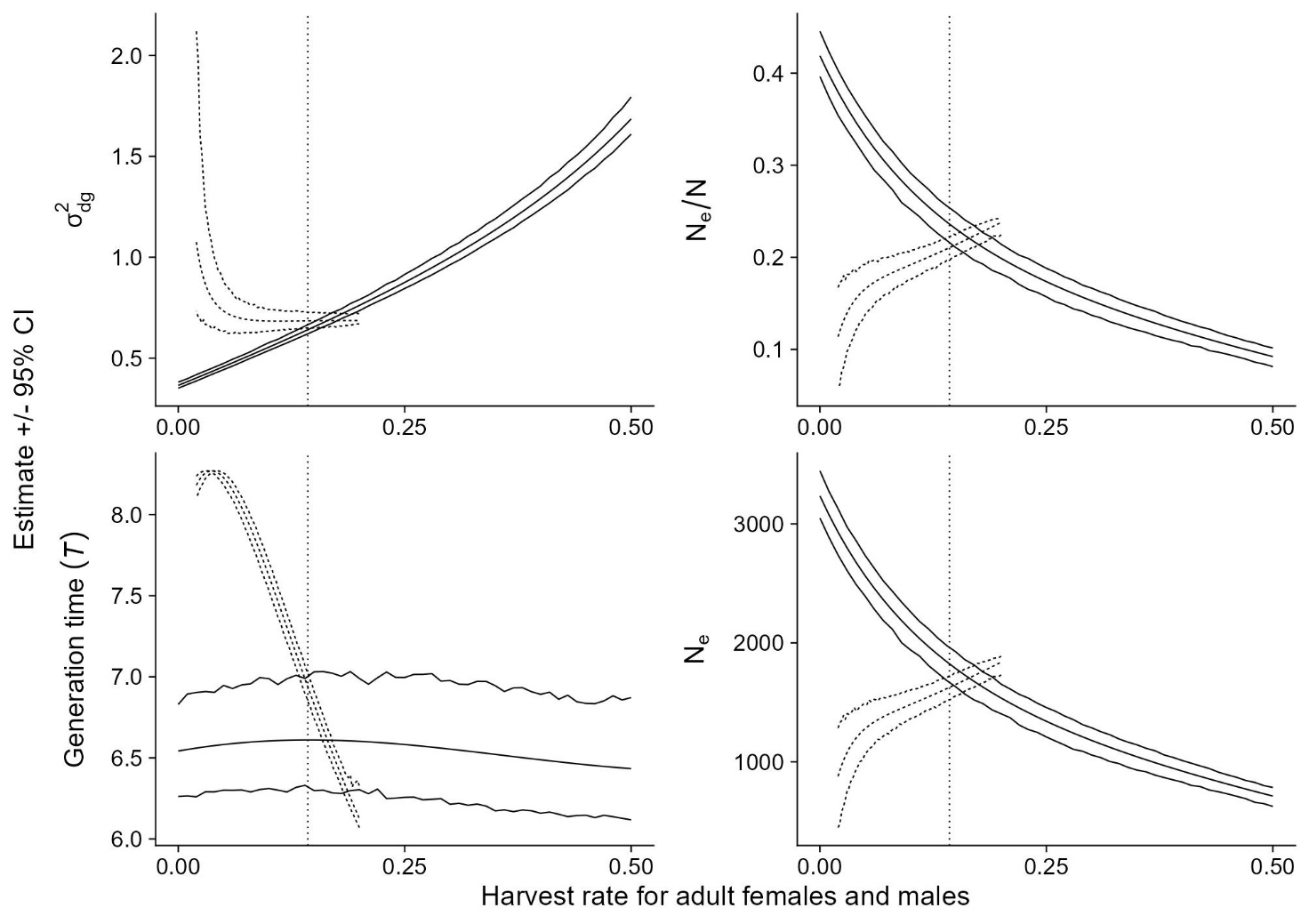


Figure C2: Demographic variance ($\sigma_{dg}^{2}$), generation time (T), ratio of effective to census population size (N_e_/N) and N_e_ (given N = 7725 in 2021) for different harvest rates for adult females and males (2.5 years and older). The solid line shows the results when the harvest rates for calves and yearlings are kept constant at their average ($h_{f,0}=0.105, h_{f,1}=0.078, h_{m,0}=0.136, h_{m,1}=0.181$), which give a growth rate λ = 1.130 when $h_{f,2}=h_{m,2}=0$ and λ = 0.754 when $h_{f,2}=h_{m,2}=0.50$. The dashed line shows the results when the average harvest rates for calves and yearlings are scaled to λ = 1. λ = 1 is not achievable if harvest rates for adult females and males are less than 2 % or larger than 21 %. The dotted vertical line indicates parameter values at the average harvest rate for adults (${(h}_{f,2}+h_{m,2})/2=0.143$).


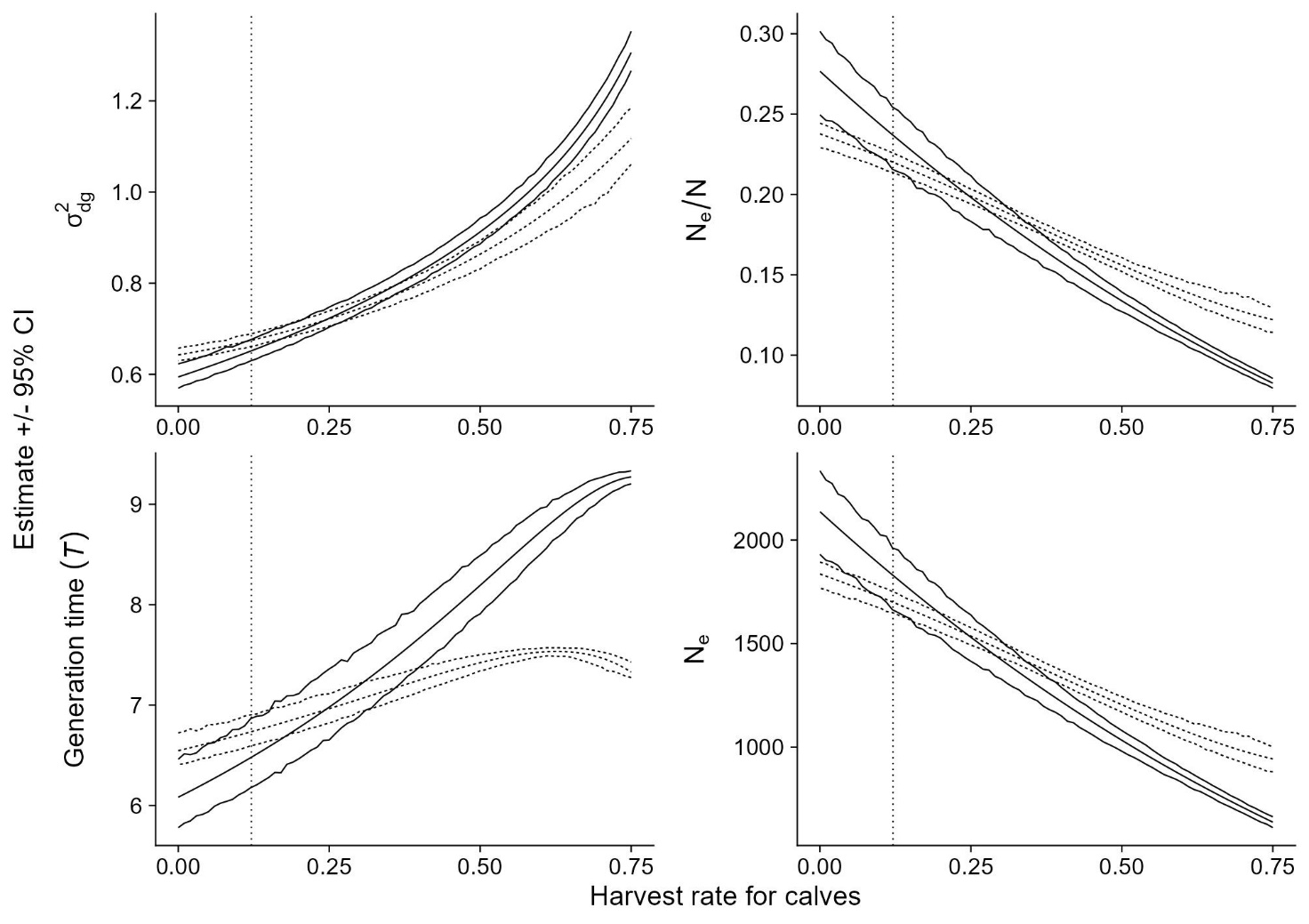


Figure C3: Demographic variance ($\sigma_{dg}^{2}$), generation time (T), ratio of effective to census population size (N_e_/N) and N_e_ (given N = 7725 in 2021) for different harvest rates for reindeer calves. The solid line shows the results when the harvest rates of yearlings and adults are kept constant at their average ($h_{f,1}=0.078, h_{f,2}=0.129, h_{m,1}=0.181, h_{m,2}=0.156$), which give growth rates λ = 1.048 when $h_{f,0}=h_{m,0}=0$ and λ = 0.884 when $h_{f,0}=h_{m,0}=0.75$. The dashed line shows the results when the average harvest rate of yearlings of both sexes and adult females are scaled to λ = 1, while the harvest rate of adult males is kept constant at its average ($h_{m,2}=0.156$). The dotted vertical line indicates the parameter values at the average harvest rate for calves (${(h}_{f,0}+h_{m,0})/2=0.121$).


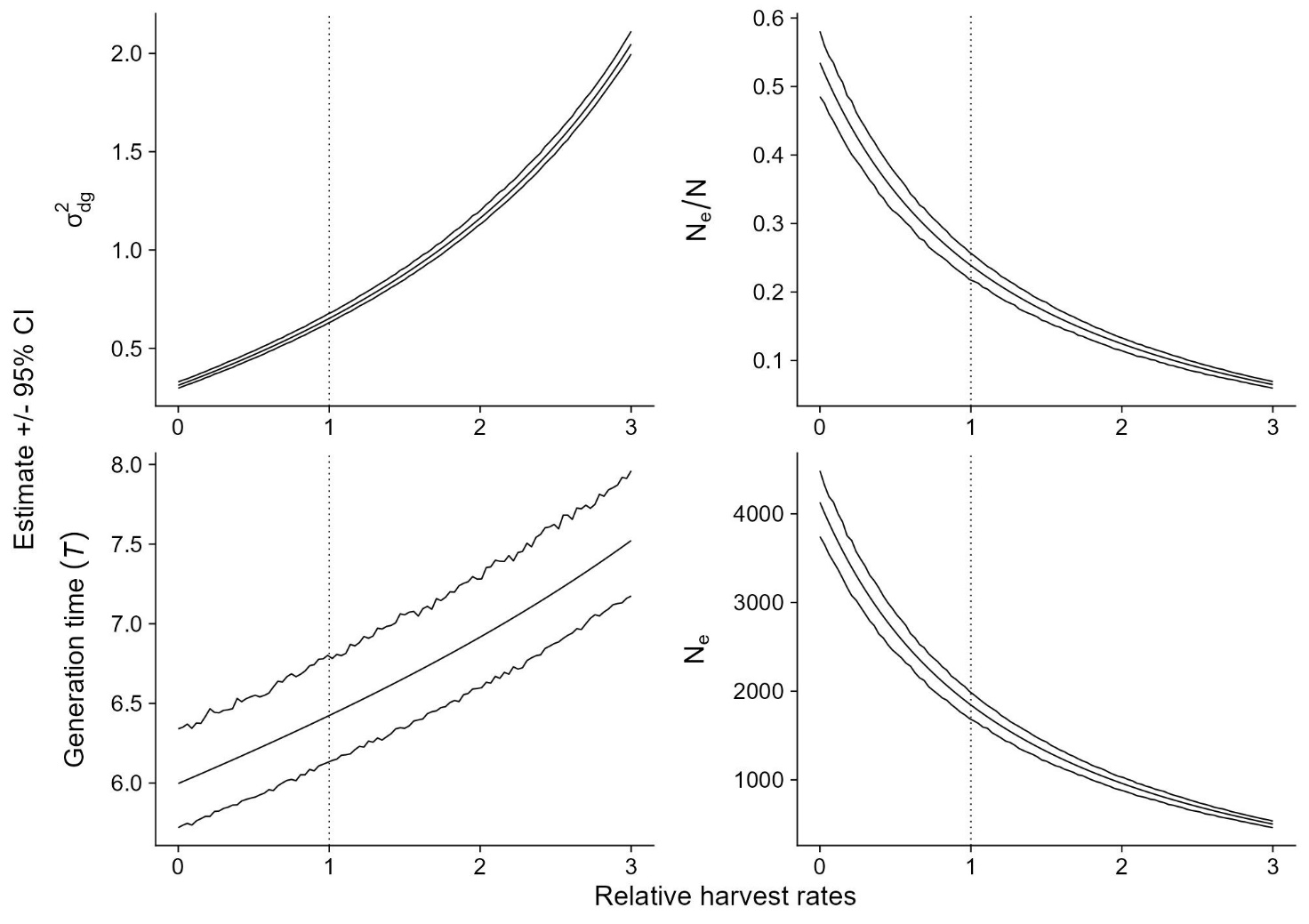


Figure C4: Demographic variance ($\sigma_{dg}^{2}$), generation time (T), ratio of effective to census population size (N_e_/N) and N_e_ (given N = 7725 in 2021) for different relative harvest rates for reindeer. The harvest rates are given relative the average rates, such that relative harvest rates of 2 equals a doubling of the average harvest rates. The growth rate λ = 1.163 when the relative harvest rates are set to 0 (no harvest) and λ = 0.757 when the relative harvest rates are set to 3. The dotted vertical line indicates the parameter values at the average harvest rates ($h_{f,0}=0.105, h_{f,1}=0.078, h_{f,2}=0.129, h_{m,0}=0.136, h_{m,1}=0.181, h_{m,2}=0.156$).


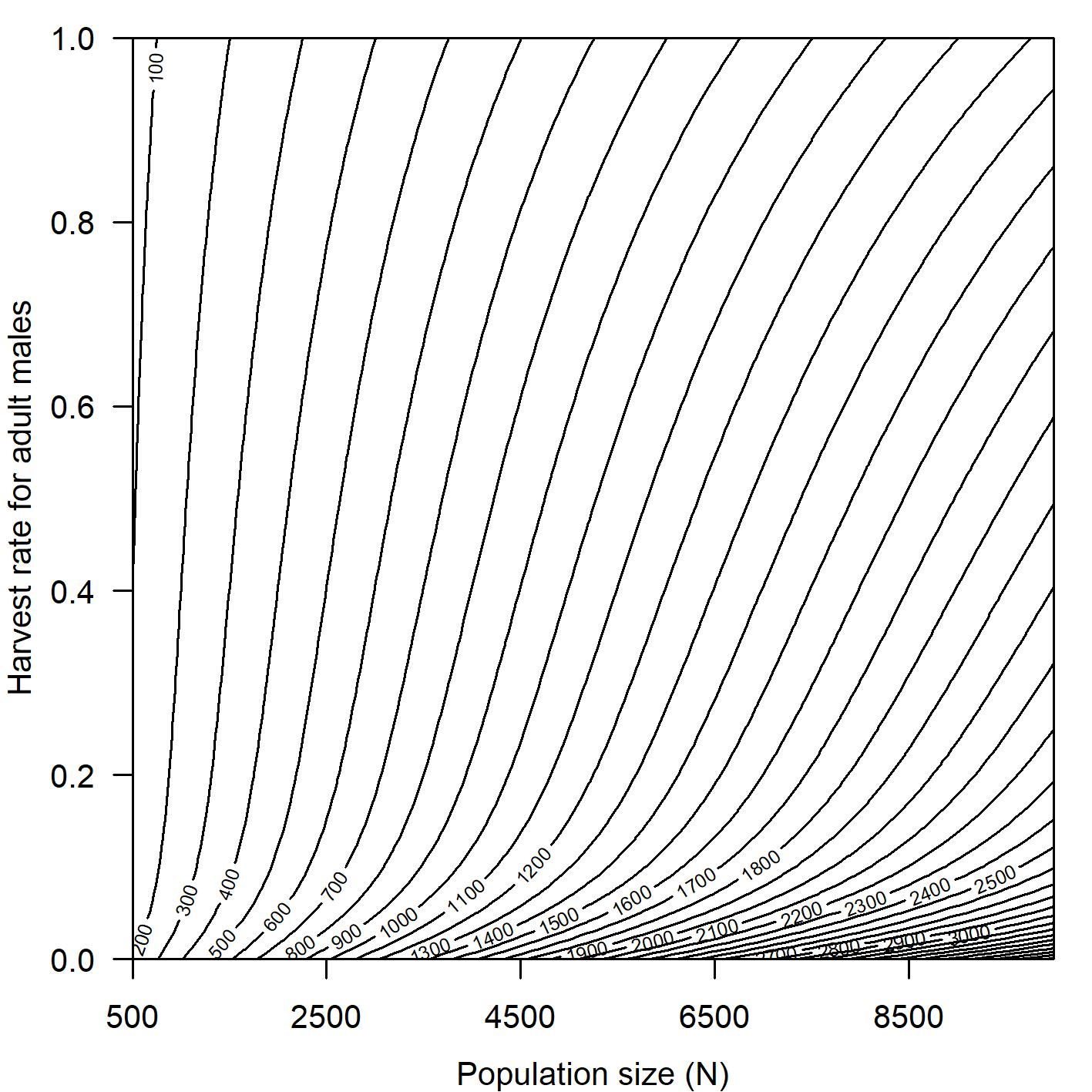


Figure C5: The effective population size (N_e_) for reindeer for the combination of census population size (N) and the harvest rate on adult males (2.5 years or older). Harvest rates for all other age and sex classes are kept constant at their average ($h_{f,0}=0.105, h_{f,1}=0.078, h_{f,2}=0.129, h_{m,0}=0.136, h_{m,1}=0.181$), which give a growth rate λ = 1.028. The post-breeding age and sex distribution of the population before harvest is set equal to the estimated distribution in 2021 ($U_{f,0}=0.125, U_{f,1}=0.087, U_{f,2}=0.430, U_{m,0}=0.125, U_{m,1}=0.085, U_{m,2}=0.151$).


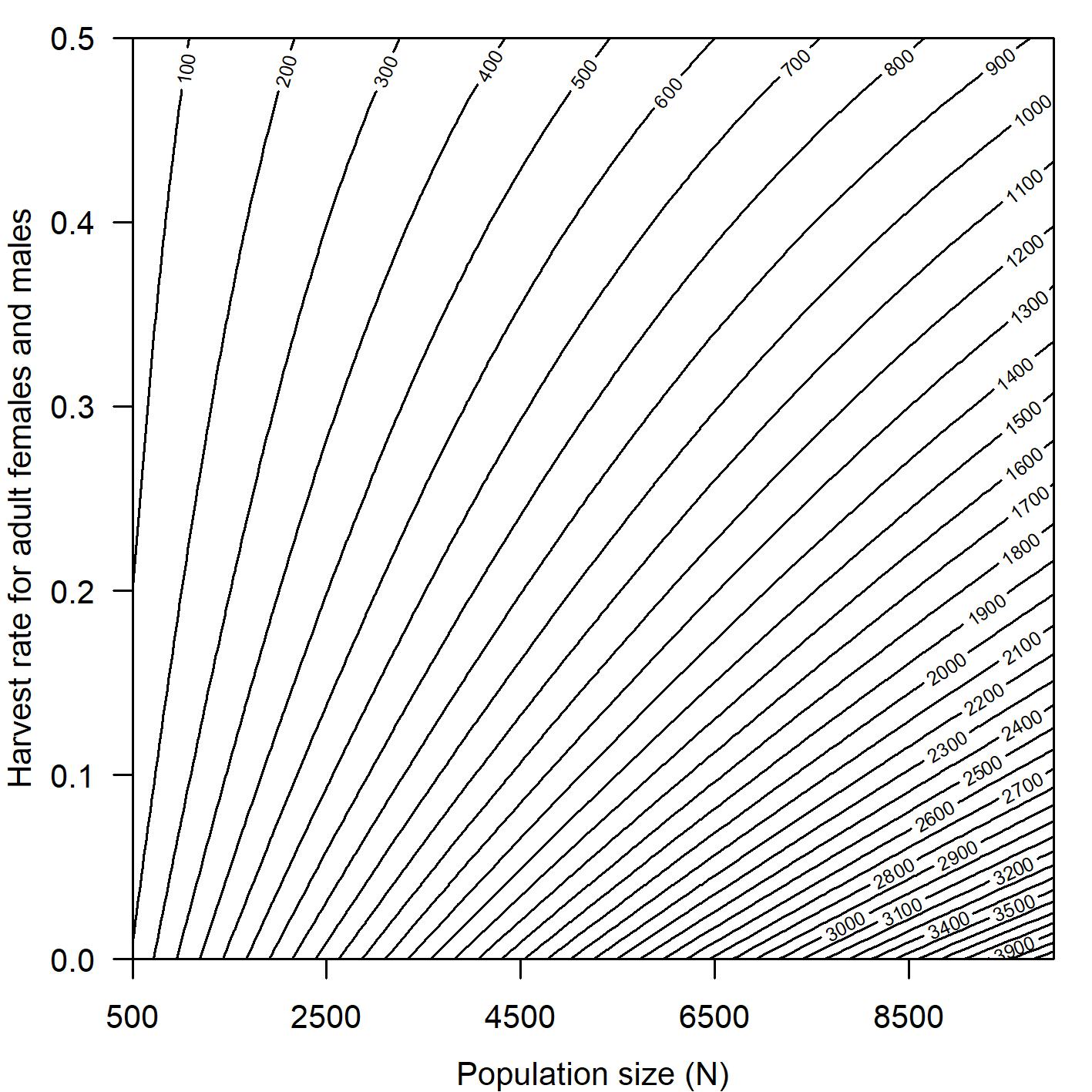


Figure C6: The effective population size (N_e_) in reindeer for the combination of census population size (N) and the harvest rate in adult females and males. Harvest rate for calves and yearlings are kept constant at their average ($h_{f,0}= 0.105, h_{f,1}=0.078, h_{m,0}=0.136, h_{m,1}=0.181$), which give a growth rate λ = 1.130 when $h_{f,2}=h_{m,2}=0$ and λ = 0.754 when $h_{f,2}=h_{m,2}=0.50$. The post-breeding age and sex distribution of the population before harvest is set equal to the estimated distribution in 2021 ($U_{f,0}=0.125, U_{f,1}=0.087, U_{f,2}=0.427, U_{m,0}=0.125, U_{m,1}=0.085, U_{m,2}=0.151$).


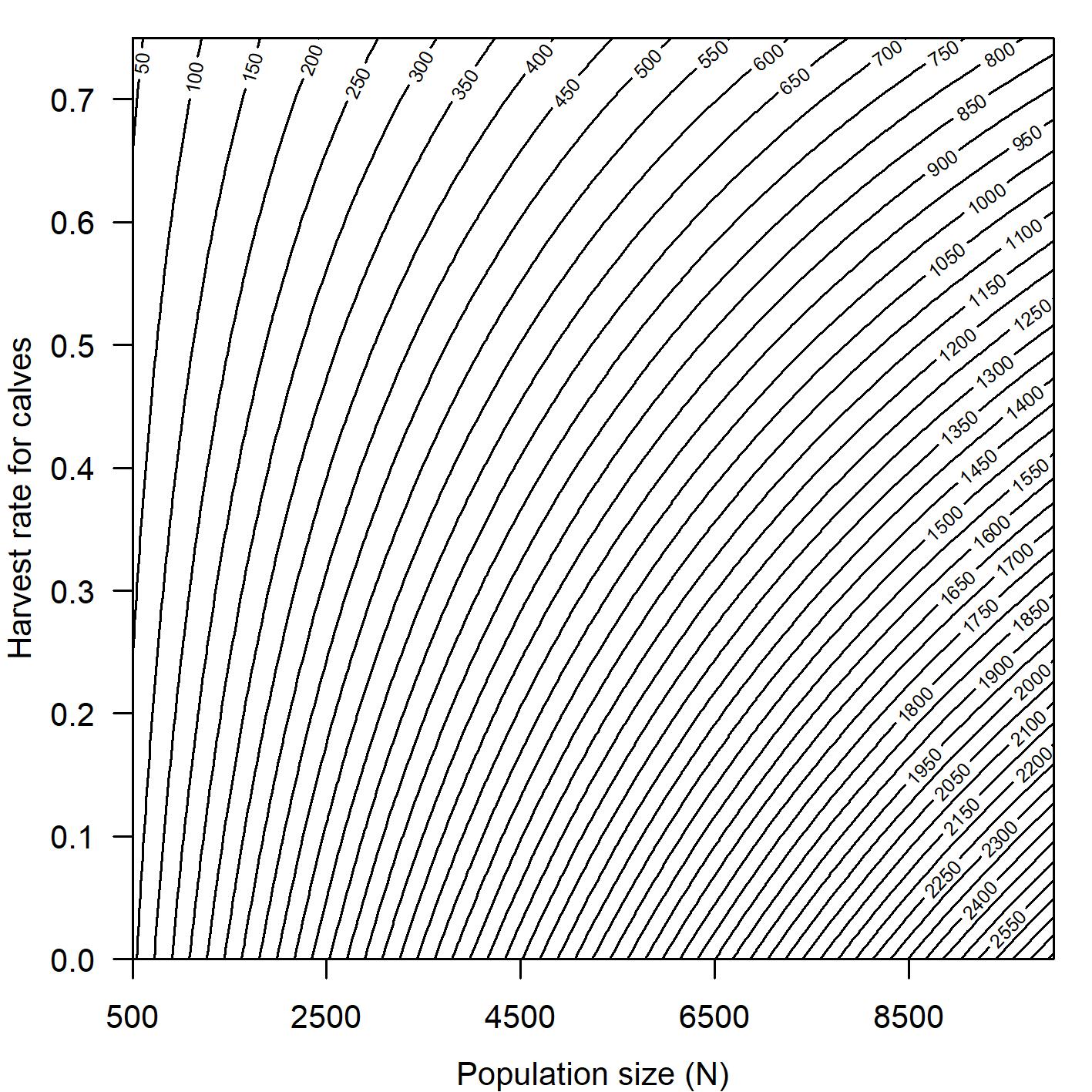


Figure C7: The effective population size (N_e_) in reindeer for the combination of census population size (N) and the harvest rate of calves. The harvest rates of yearlings and adults are kept constant at their average ($h_{f,1}=0.078, h_{f,2}=0.129, h_{m,1}=0.181, h_{m,2}=0.156$), which gives growth rates λ = 1.048 when $h_{f,0}=h_{m,0}=0$ and λ = 0.884 when $h_{f,0}=h_{m,0}=0.75$. The post-breeding age and sex distribution of the population prior to harvest is set equal to the estimated distribution in 2021 ($U_{f,0}=0.125, U_{f,1}=0.087, U_{f,2}=0.427, U_{m,0}=0.125, U_{m,1}=0.085, U_{m,2}=0.151$).


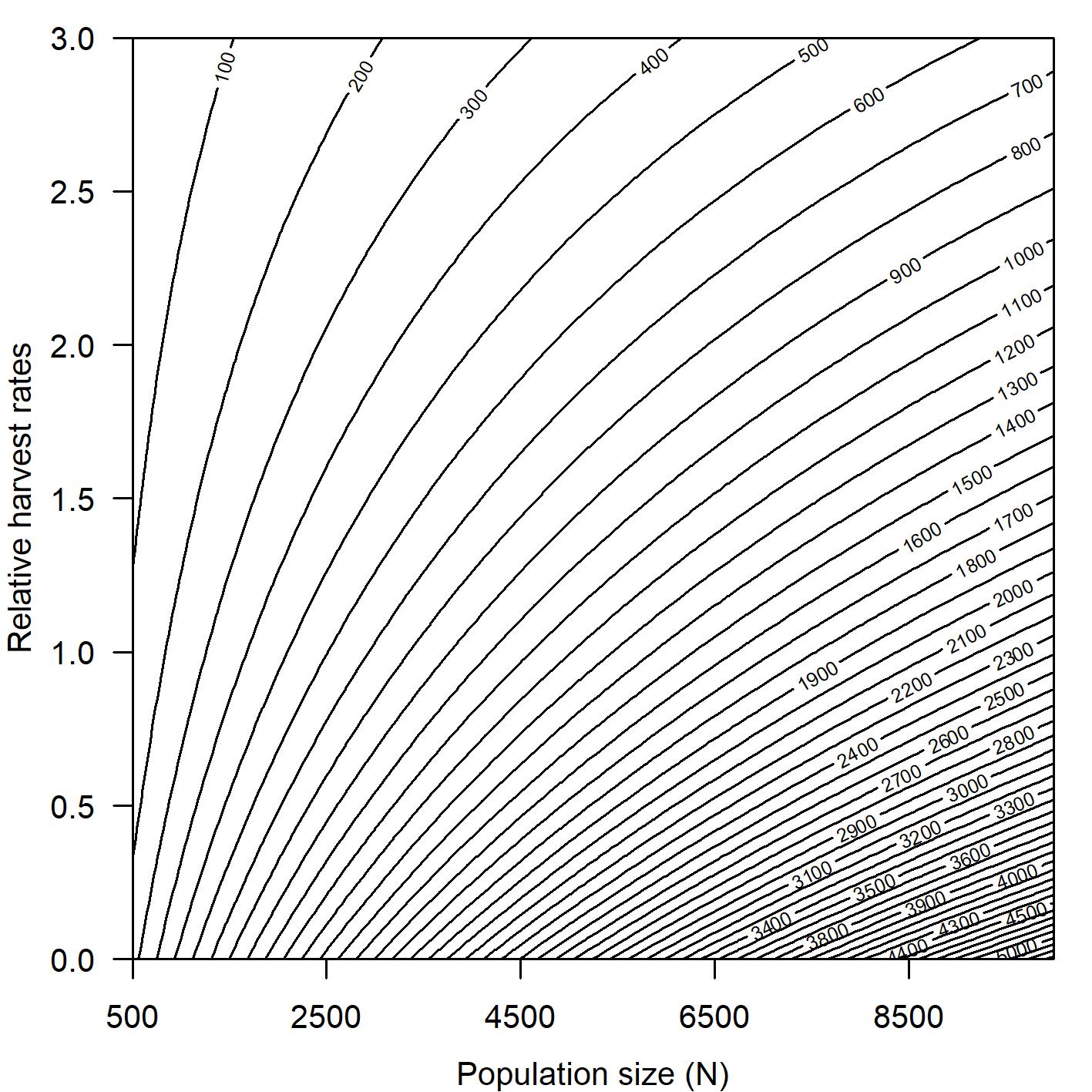


Figure C8: The effective population size (N_e_) for reindeer for combinations of census population size (N) and the relative harvest rate. The harvest rates are given relative to the average rates, such that a relative harvest rate of 2 equals a doubling of the average harvest rates. The growth rate λ = 1.163 when the relative harvest rates are set to 0 (no harvest) and λ = 0.757 when the relative harvest rates are set to 3. The post-breeding age and sex distribution of the population before harvest is set equal to the estimated distribution in 2021 ($U_{f,0}=0.125, U_{f,1}=0.087, U_{f,2}=0.427, U_{m,0}=0.125, U_{m,1}=0.085, U_{m,2}=0.151$).

## D. Simulations of genetic drift - Temporal change in population size and heterozygosity


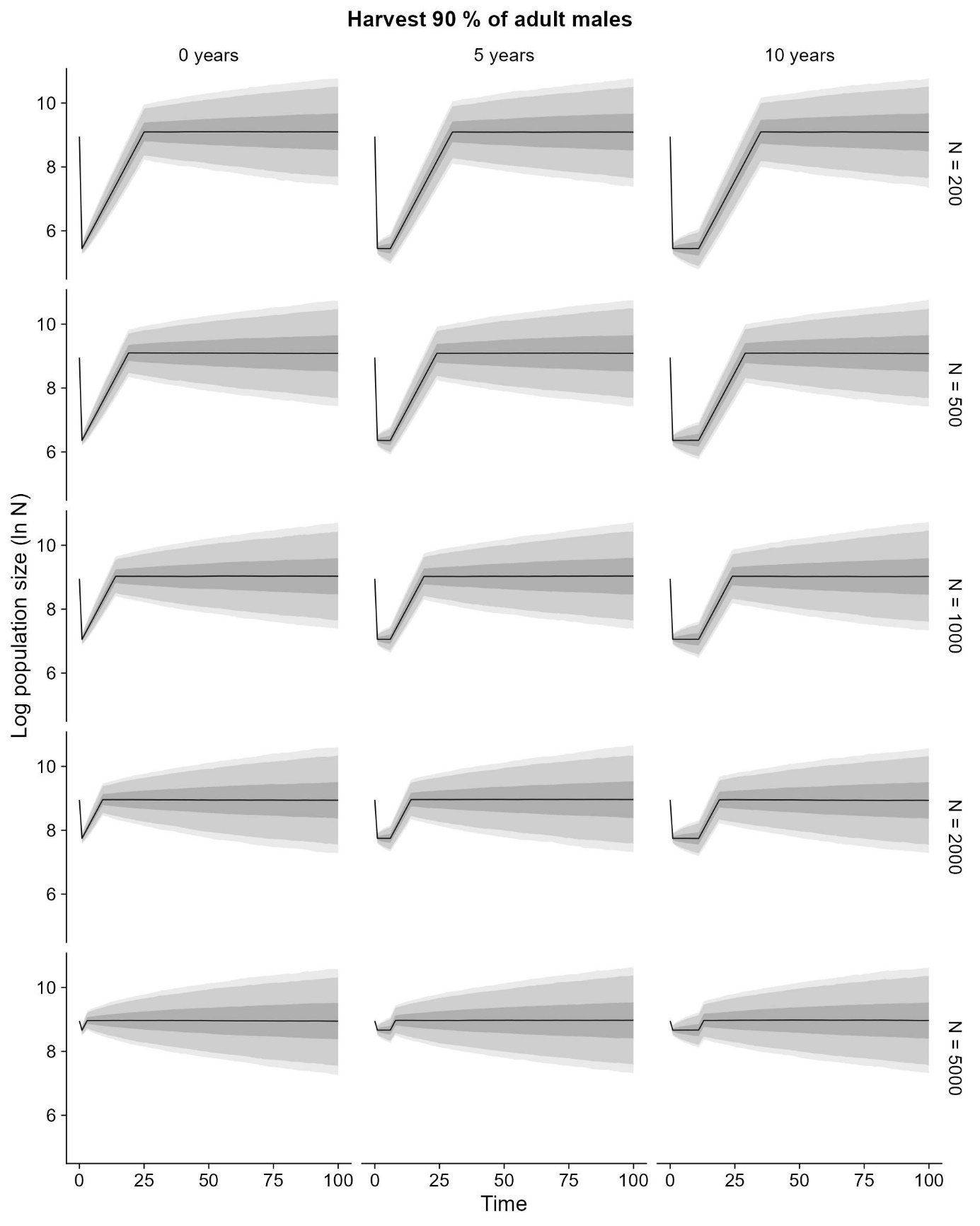


Figure D1: The distribution of ln N over time in simulations with a population decimation where the population is kept at the reduced population size for 0, 5 or 10 years. 90 % of adult males are harvested while the population is kept low and stable population dynamics are achieved by scaling the average harvest rates of calves, yearlings and adult females. N_0_ = 7725, $\sigma_{d}^{2}$ = 0.575 and $\sigma_{e}^{2}$ = 0.007. After the period with reduced population size the population is allowed to grow without harvest until it reaches N_0_ and are kept stable there by scaling all the average harvest rates ($h_{f,0}=0.105, h_{f,1}=0.078, h_{f,2}=0.129, h_{m,0}=0.136, h_{m,1}=0.181, h_{m,2}=0.156$). The solid line shows the average, while 50, 90 and 95 % confidence intervals are shown in different shades of grey.


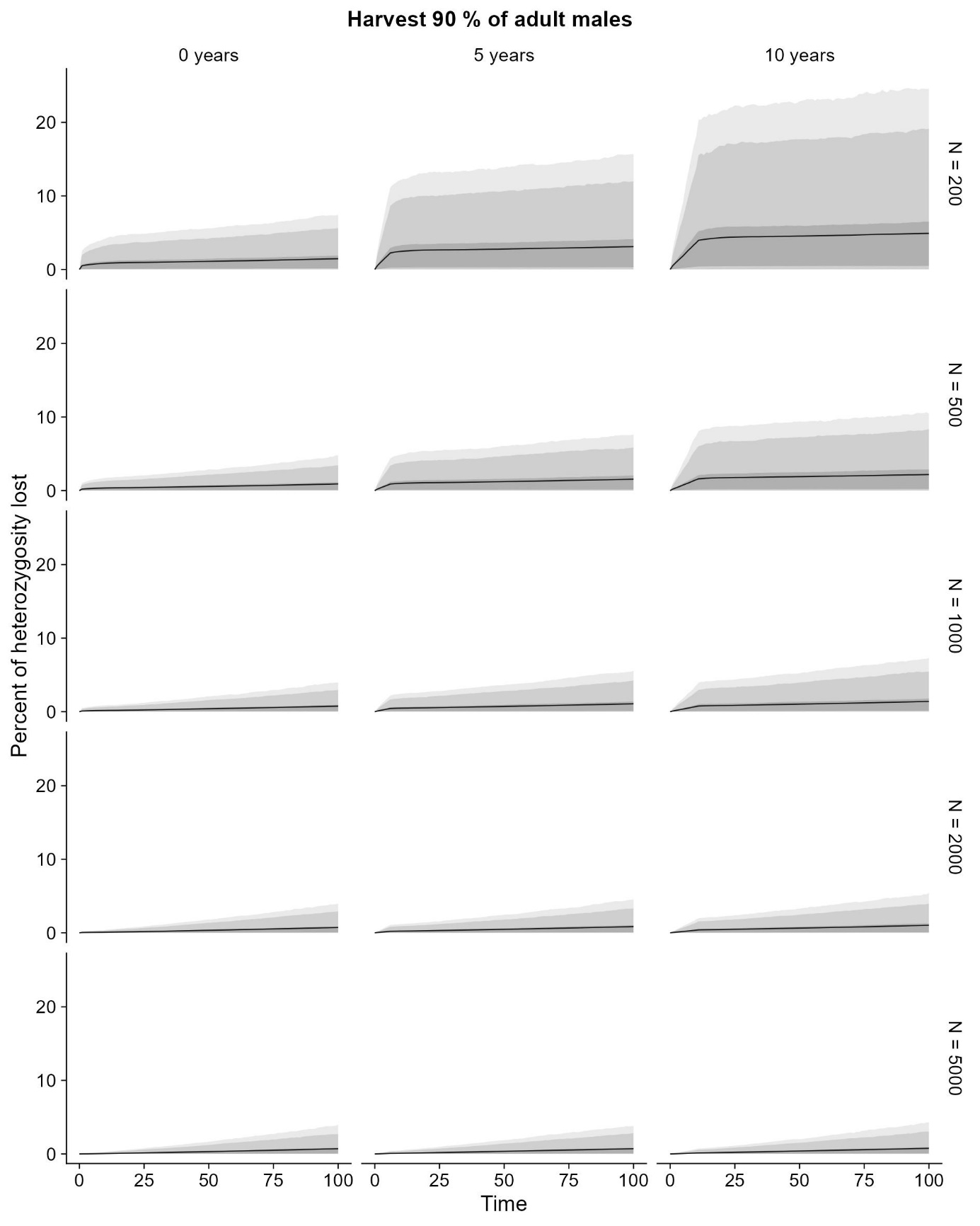


Figure D2: The distribution of the loss of heterozygosity over time in simulations with a population decimation where the population is kept at the reduced population size for 0, 5 or 10 years. 90 % of adult males are harvested while the population is kept low and stable population dynamics are achieved by scaling the average harvest rates of calves, yearlings and adult females. N_0_ = 7725, $\sigma_{d}^{2}$ = 0.575 and $\sigma_{e}^{2}$ = 0.007. After the period with reduced population size the population is allowed to grow without harvest until it reaches N_0_ and are kept stable there by scaling all the average harvest rates ($h_{f,0}=0.105, h_{f,1}=0.078, h_{f,2}=0.129, h_{m,0}=0.136, h_{m,1}=0.181, h_{m,2}=0.156$). The amount of genetic drift in the population change in accordance with changes in the population size and harvest rates. The solid line shows the average, while 50, 90 and 95 % confidence intervals are shown in different shades of grey.


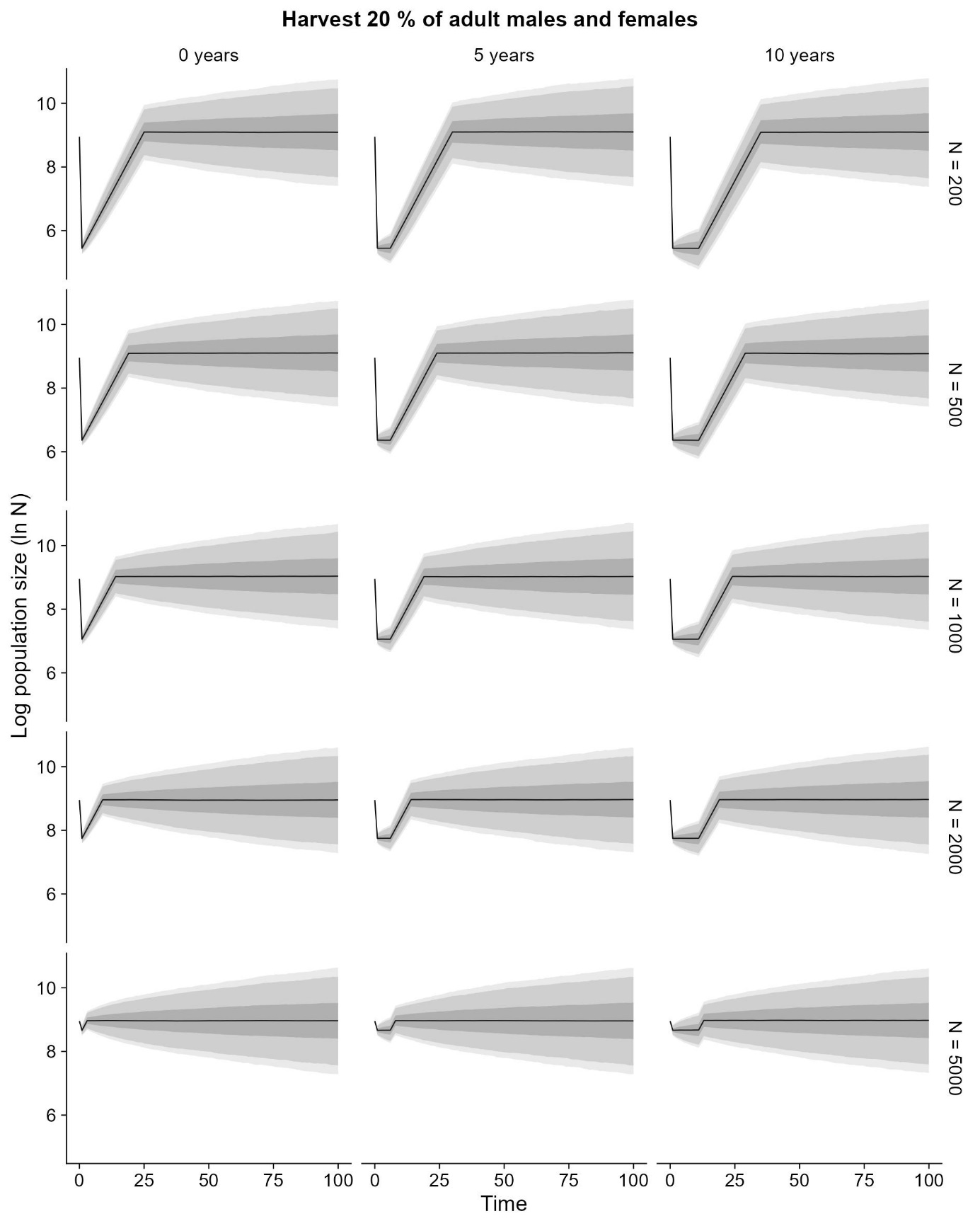


Figure D3: The distribution of ln N over time in simulations with a population decimation where the population is kept at the reduced population size for 0, 5 or 10 years. 20 % of adult males and females are harvested while the population is kept low and stable population dynamics are achieved by scaling the average harvest rates of calves and yearlings. N_0_ = 7725, $\sigma_{d}^{2}$ = 0.575 and $\sigma_{e}^{2}$ = 0.007. After the period with reduced population size the population is allowed to grow without harvest until it reaches N_0_ and are kept stable there by scaling all the average harvest rates ($h_{f,0}=0.105, h_{f,1}=0.078, h_{f,2}=0.129, h_{m,0}=0.136, h_{m,1}=0.181, h_{m,2}=0.156$). The solid line shows the average, while 50, 90 and 95 % confidence intervals are shown in different shades of grey.


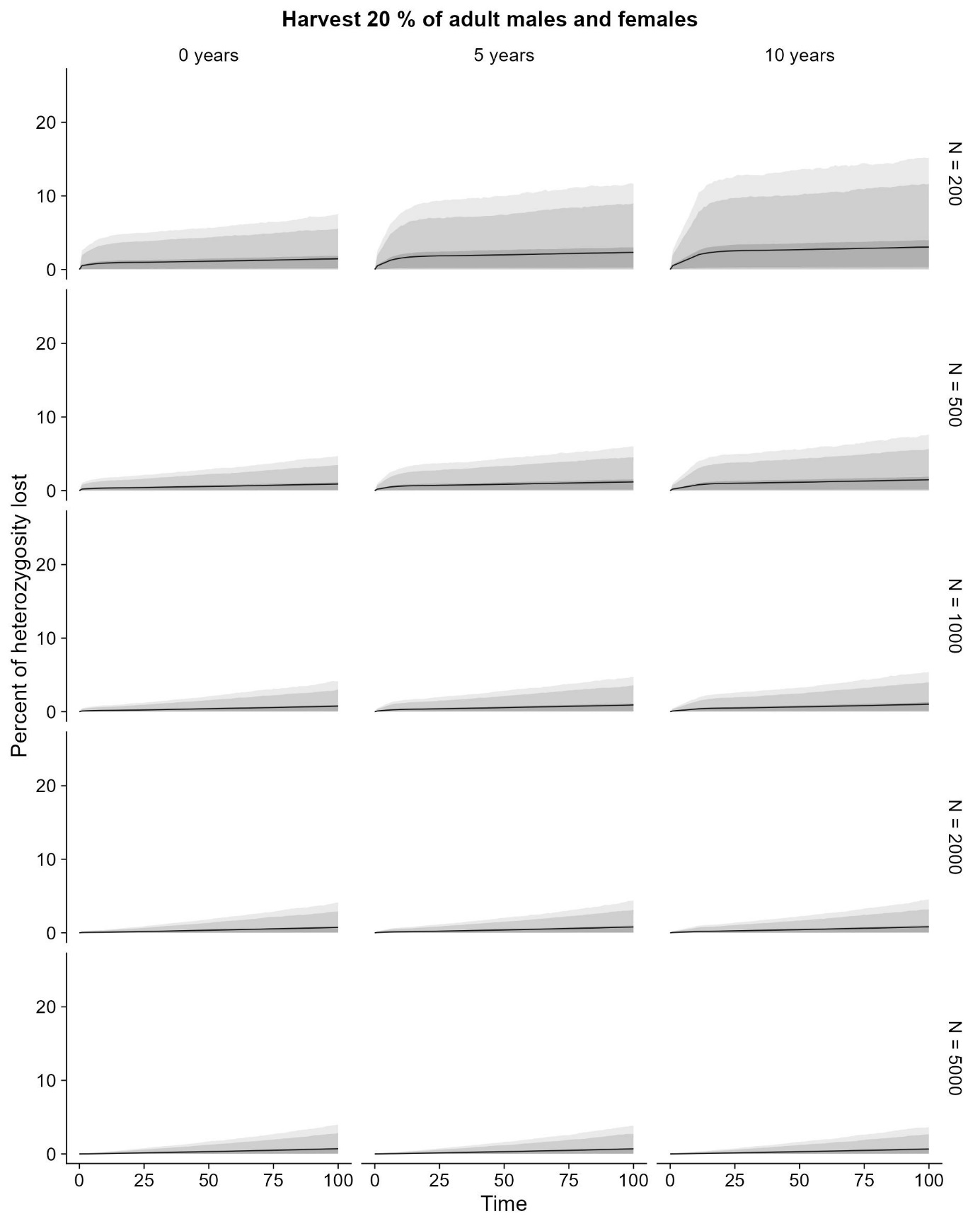


Figure D4: The distribution of the loss of heterozygosity over time in simulations with a population decimation where the population is kept at the reduced population size for 0, 5 or 10 years. 20 % of adult males and females are harvested while the population is kept low and stable population dynamics are achieved by scaling the average harvest rates of calves and yearlings. N_0_ = 7725, $\sigma_{d}^{2}$ = 0.575 and $\sigma_{e}^{2}$ = 0.007. After the period with reduced population size the population is allowed to grow without harvest until it reaches N_0_ and are kept stable there by scaling all the average harvest rates ($h_{f,0}=0.105, h_{f,1}=0.078, h_{f,2}=0.129, h_{m,0}=0.136, h_{m,1}=0.181, h_{m,2}=0.156$). The amount of genetic drift in the population change in accordance with changes in the population size and harvest rates. The solid line shows the average, while 50, 90 and 95 % confidence intervals are shown in different shades of grey.


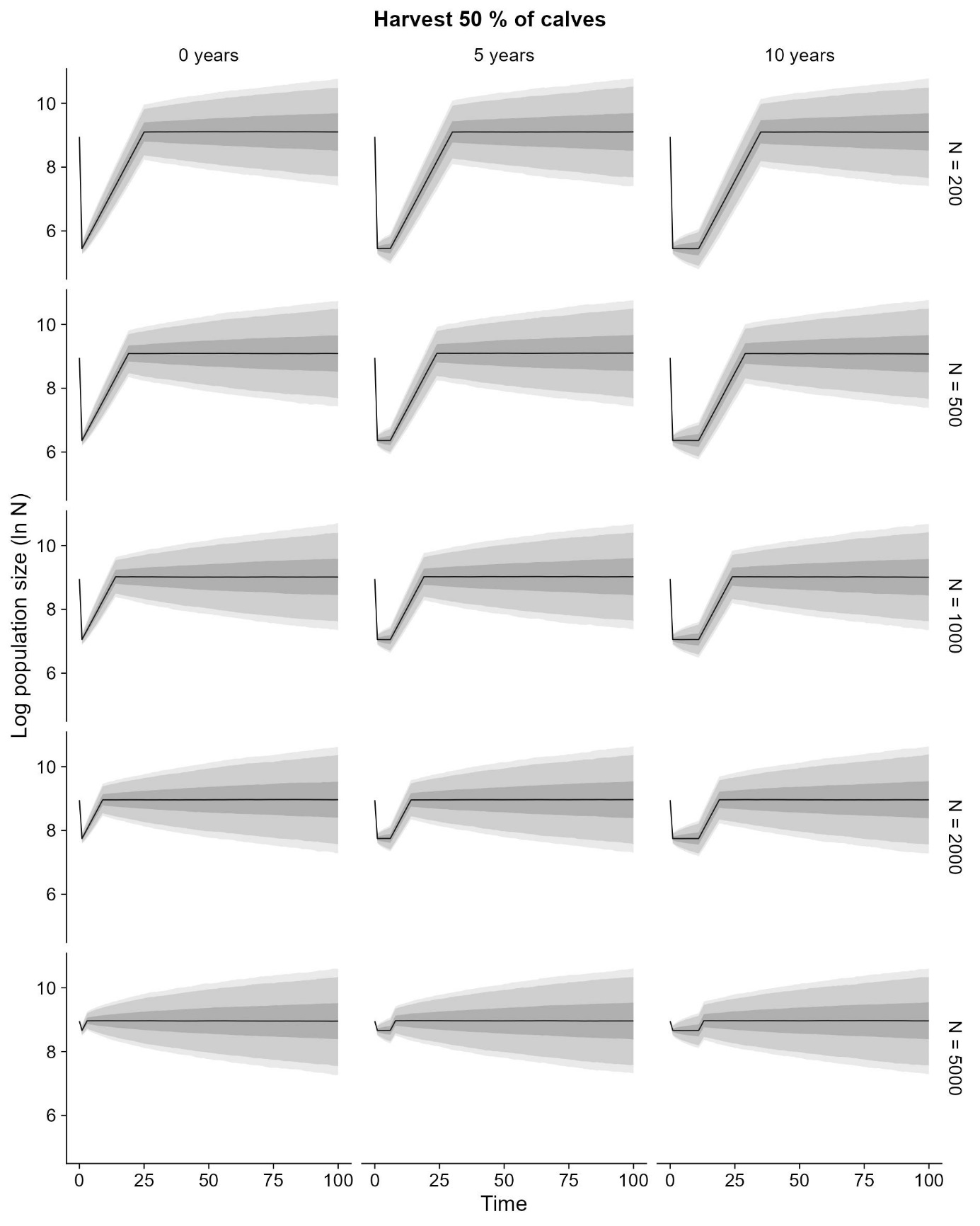


Figure D5: The distribution of ln N over time in simulations with a population decimation where the population is kept at the reduced population size for 0, 5 or 10 years. 50 % of calves are harvested while the population is kept low and stable population dynamics are achieved by scaling the average harvest rates of yearlings and adult females. N_0_ = 7725, $\sigma_{d}^{2}$ = 0.575 and $\sigma_{e}^{2}$ = 0.007. After the period with reduced population size the population is allowed to grow without harvest until it reaches N_0_ and are kept stable there by scaling all the average harvest rates ($h_{f,0}=0.105, h_{f,1}=0.078, h_{f,2}=0.129, h_{m,0}=0.136, h_{m,1}=0.181, h_{m,2}=0.156$). The solid line shows the average, while 50, 90 and 95 % confidence intervals are shown in different shades of grey.


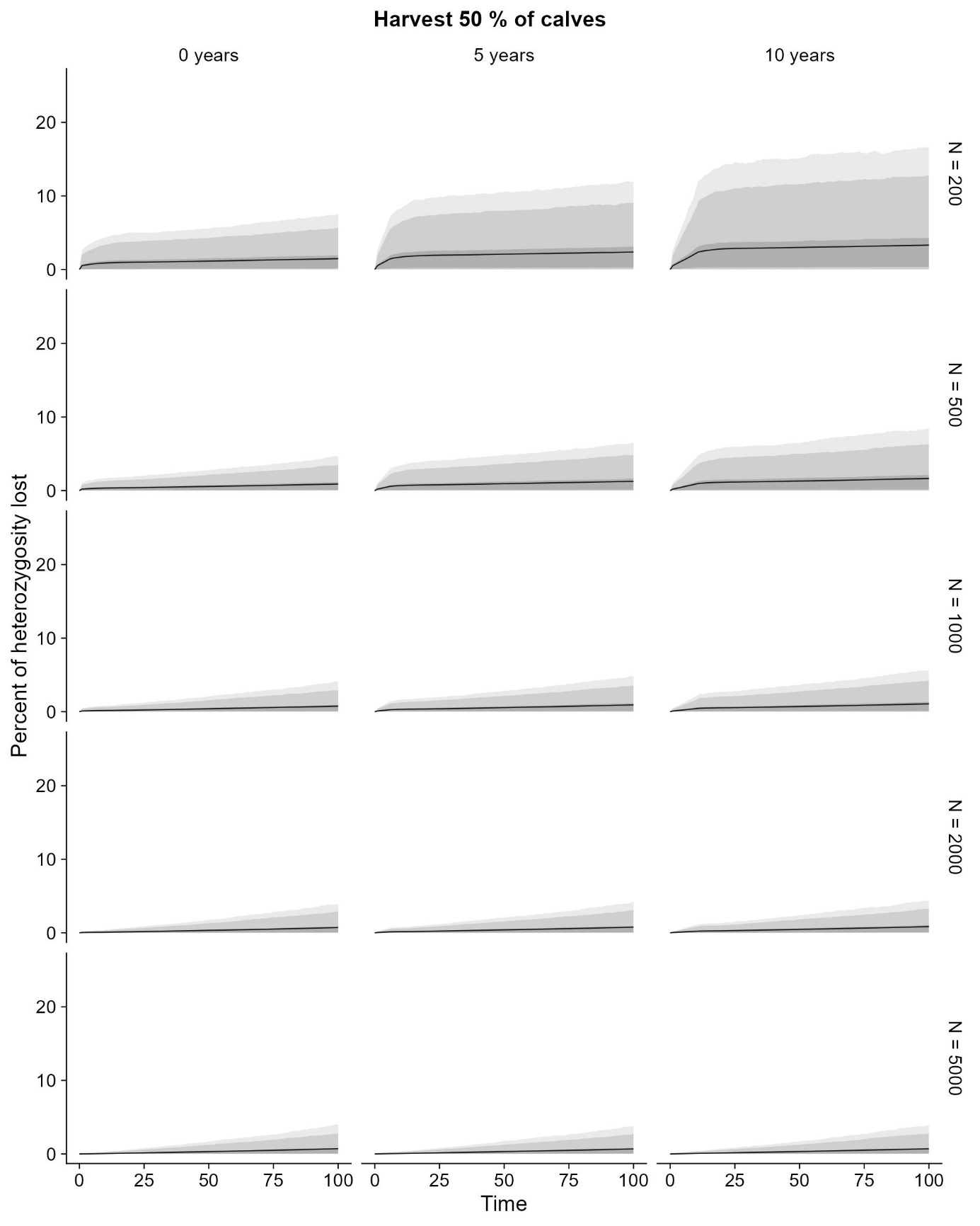


Figure D6: The distribution of the loss of heterozygosity over time in simulations with a population decimation where the population is kept at the reduced population size for 0, 5 or 10 years. 50 % of calves are harvested while the population is kept low and stable population dynamics are achieved by scaling the average harvest rates of yearlings and adult females. N_0_ = 7725, $\sigma_{d}^{2}$ = 0.575 and $\sigma_{e}^{2}$ = 0.007. After the period with reduced population size the population is allowed to grow without harvest until it reaches N_0_ and are kept stable there by scaling all the average harvest rates ($h_{f,0}=0.105, h_{f,1}=0.078, h_{f,2}=0.129, h_{m,0}=0.136, h_{m,1}=0.181, h_{m,2}=0.156$). The amount of genetic drift in the population change in accordance with changes in the population size and harvest rates. The solid line shows the average, while 50, 90 and 95 % confidence intervals are shown in different shades of grey.


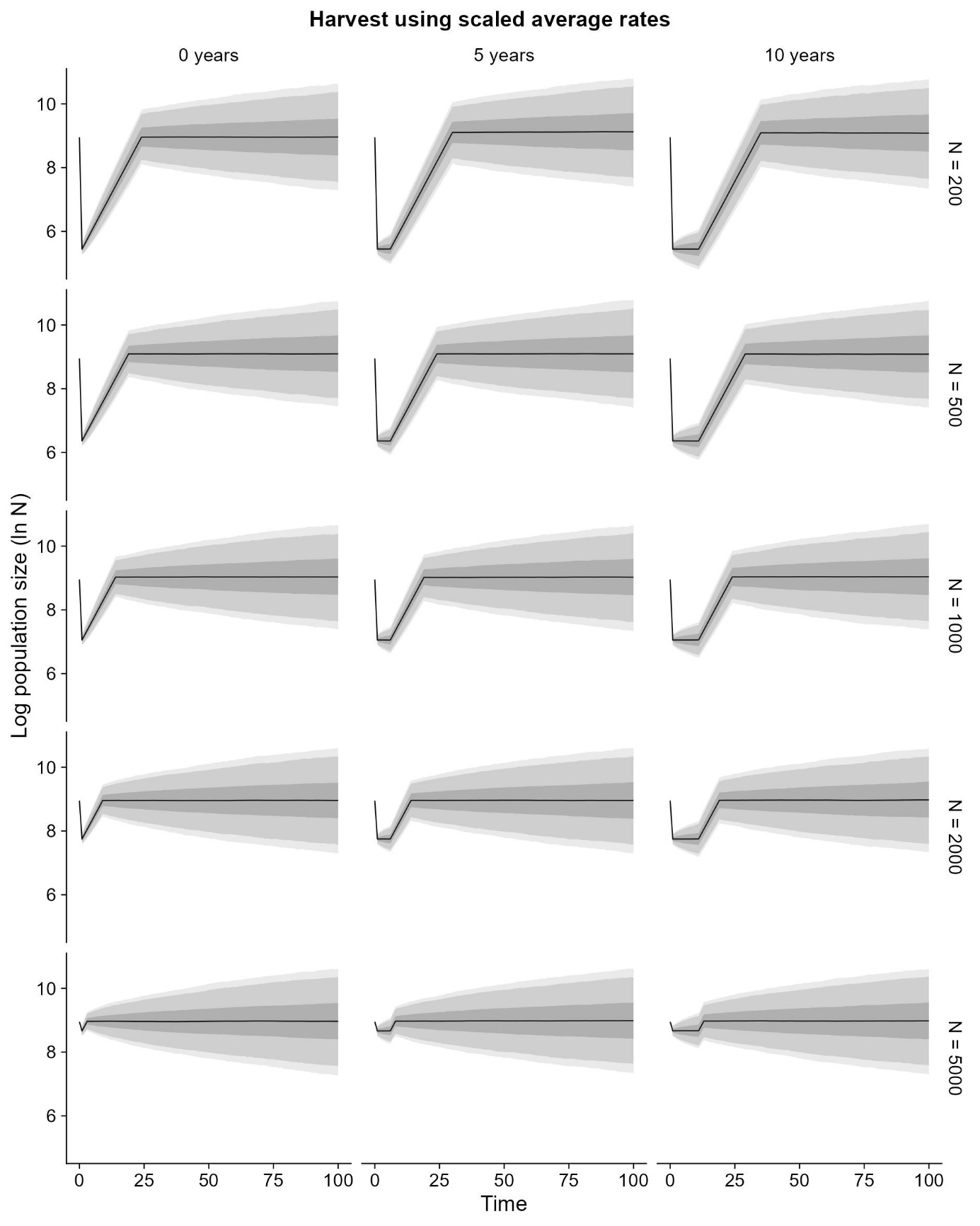


Figure D7: The distribution of ln N over time in simulations with a population decimation where the population is kept at the reduced population size for 0, 5 or 10 years. The population is harvested with scaled average rates while the population is kept low. N_0_ = 7725, $\sigma_{d}^{2}$ = 0.575 and $\sigma_{e}^{2}$ = 0.007. After the period with reduced population size the population is allowed to grow without harvest until it reaches N_0_ and are kept stable there by scaling all the average harvest rates ($h_{f,0}=0.105, h_{f,1}=0.078, h_{f,2}=0.129, h_{m,0}=0.136, h_{m,1}=0.181, h_{m,2}=0.156$). The solid line shows the average, while 50, 90 and 95 % confidence intervals are shown in different shades of grey.


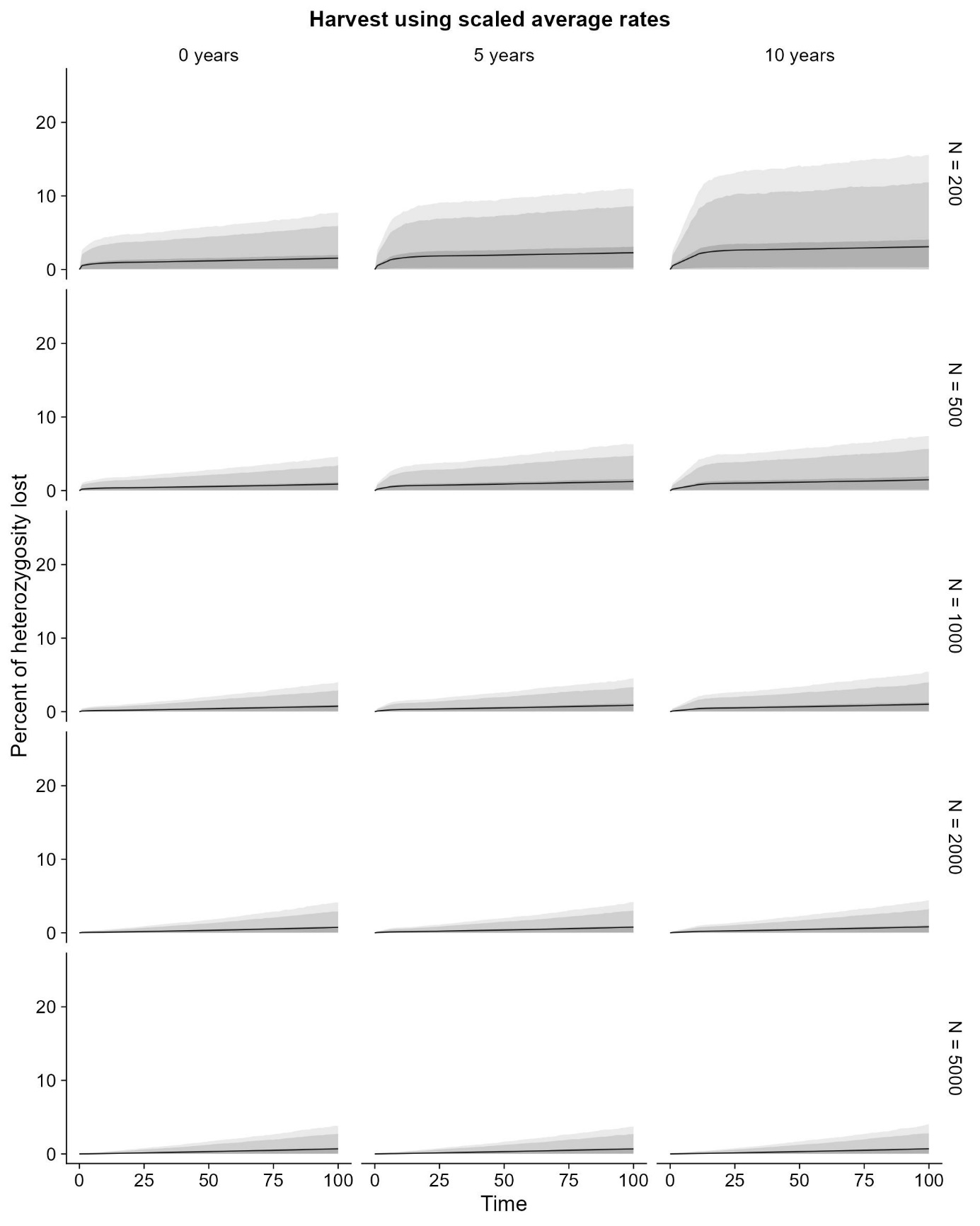


Figure D8: The distribution of the loss of heterozygosity over time in simulations with a population decimation where the population is kept at the reduced population size for 0, 5 or 10 years. The population is harvested with scaled average rates while the population is kept low. N_0_ = 7725, $\sigma_{d}^{2}$ = 0.575 and $\sigma_{e}^{2}$ = 0.007. After the period with reduced population size the population is allowed to grow without harvest until it reaches N_0_ and are kept stable there by scaling all the average harvest rates ($h_{f,0}=0.105, h_{f,1}=0.078, h_{f,2}=0.129, h_{m,0}=0.136, h_{m,1}=0.181, h_{m,2}=0.156$). The amount of genetic drift in the population change in accordance with changes in the population size and harvest rates. The solid line shows the average, while 50, 90 and 95 % confidence intervals are shown in different shades of grey.
